# ^1^H HR-MAS NMR-Based Metabolomics of Cancer Cells in Response to Treatment with the Diruthenium Trithiolato Complex [(*p*-MeC_6_H_4_*^i^*Pr)_2_Ru_2_(SC_6_H_4_-*p*-Bu*^t^*)_3_]^+^ (DiRu-1)

**DOI:** 10.3390/metabo9070146

**Published:** 2019-07-18

**Authors:** Hedvika Primasová, Lydia E. H. Paul, Gaëlle Diserens, Ester Primasová, Peter Vermathen, Martina Vermathen, Julien Furrer

**Affiliations:** 1 Department of Chemistry and Biochemistry, University of Bern, Freiestrasse 3, 3012 Bern, Switzerland; 2 Department of BioMedical Research and Radiology, University of Bern and Inselspital, Erlachstrasse 9a, 3012 Bern, Switzerland; 3 Faculty of Information Technology, Czech Technical University in Prague, Thákurova 9, 16000 Prague, Czech Republic

**Keywords:** ovarian cancer, cytotoxicity, ruthenium complex, HR-MAS NMR, NMR metabolomics, A2780, cis-Pt resistant, metal-based drugs

## Abstract

The trithiolato bridged diruthenium complex DiRu-1 [(*p*-MeC_6_H_4_*^i^*Pr)_2_Ru_2_(SC_6_H_4_-*p*-Bu*^t^*)_3_]^+^ is highly cytotoxic against various cancer cell lines, but its exact mode of action remains unknown. The present ^1^H HR-MAS NMR-based metabolomic study was performed on ovarian cancer cell line A2780, on its cis-Pt resistant variant A2780cisR, and on the cell line HEK-293 treated with 0.03 µM and 0.015 µM of DiRu-1 corresponding to full and half IC_50_ doses, respectively, to investigate the mode of action of this ruthenium complex. The resulting changes in the metabolic profile of the cell lines were studied using HR-MAS NMR of cell lysates and a subsequent statistical analysis. We show that DiRu-1 in a 0.03 µM dose has significant impact on the levels of a number of metabolites, such as glutamine, glutamate, glutathione, cysteine, lipid, creatine, lactate, and acetate, especially pronounced in the A2780cisR cell line. The IC_50_/2 dose shows some significant changes, but full IC_50_ appears to be necessary to observe the full effect. Overall, the metabolic changes observed suggest that redox homeostasis, the Warburg effect, and the lipid metabolism are affected by DiRu-1.

## 1. Introduction

Fifty years after the discovery of its antiproliferative properties, Cis-platin (Cis-Pt) is the most used metal-based drug in cancer treatment. It has been used over decades for the treatment of ovarian, testicular, and other types of cancer and is still used today in combination with other drugs in more than 50% of all chemotherapies [1]. The numerous side effects and the emergence of resistance have encouraged further development of platinum compounds as well as of other metal-based compounds as anticancer drug candidates. Ruthenium-based complexes belong to the most promising ones [1,2]. Two ruthenium (III) compounds, NAMI-A and NKP1339, have entered clinical tests recently [3]. Half-sandwich arene ruthenium (II) complexes have also attracted attention since the discovery by Tocher et al. in 1992 that observed a cytotoxicity enhancement by coordinating the anticancer agent metronidazole to a benzene ruthenium dichloro fragment [4]. Out of the numerous arene ruthenium (II) compounds synthesized and evaluated so far, RAPTA-C from the RAPTA family and RM175 from the RAED [(η^6^-arene)Ru(en)Cl]+, where en = ethylenediamine) family are the most advanced. RAPTA-C has been very active in vivo, where it inhibited lung metastases in mice. RAED compounds are highly cytotoxic against a number of cancer cell lines including those that developed resistance against cis-Pt [5,6,7].

**Figure 1:**
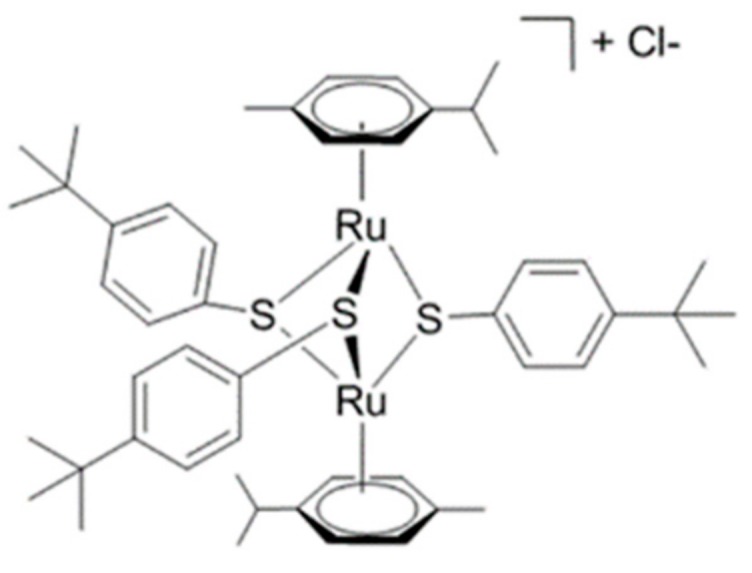
Structure of [(η ^6^-*p*-MeC_6_H_4_Pr*^i^*)_2_Ru_2_(µ _2_-SC_6_H_4_-*p*-Bu*^t^*)_3_]Cl (DiRu-1).

Our group has developed a large variety of diruthenium *p*-cymene trithiolato bridged complexes, obtained from the reaction of [(*p*-MeC_6_H_4_*^i^*Pr)_2_Ru_2_Cl_2_] with the corresponding aromatic thiols. The most active compound obtained so far is [(η^6^-*p*-MeC_6_H_4_Pr*^i^*)_2_Ru_2_(µ_2_-SC_6_H_4_-*p*-Bu*^t^*)_3_]Cl (DiRu-1) (Figure 1), with an IC_50_ of 0.03 µM towards the A2780 human ovarian cancer cell line and the cisplatin-resistant A2780cisR cell line. Additionally, DiRu-1 is highly cytotoxic against hepatocellular carcinoma (HepG2), estrogen-responsive breast adenocarcinoma (MCF-7) and triple-negative breast adenocarcinoma (MDA-MB-231) cell lines with IC_50_ values in the nanomolar range (Table 1). A recent in vivo study has demonstrated that DiRu-1 significantly prolongs the survival of tumor-bearing mice [8]. Obviously, its mode of action differs from that of platinum complexes, but the exact mechanism is currently not known. Though it was found that DiRu-1 has the ability to catalyze glutathione (GSH) oxidation, this mechanism can only explain its cytotoxicity to a certain extent. Recently, it was found that, when using inductively coupled plasma mass spectrometry (ICP-MS), 97% of the ruthenium is localized in the mitochondria of treated A2780 cells [9,10,11].

It is a matter of fact that the energy metabolism of cancer cells is altered compared to that of healthy cells. The change in metabolism manifesting as cancer cells ferment glucose to lactate even in the presence of oxygen instead of mitochondrial oxidative phosphorylation is known as the Warburg effect [12,13]. While being less efficient than mitochondrial respiration in terms of adenosine triphosphate (ATP) production, anaerobic glycolysis is a faster way to catabolize glucose. Over the same period of time, equal amounts of ATP are therefore generated in both types of glucose catabolism. While the current understanding of how cancer cells benefit from the Warburg effect is likely to be not yet complete [14], a number of possible explanations why cancer cells switch towards aerobic glycolysis have been suggested in the past. To mention a few, one explanation is that less efficient ATP production is only a problem when there is a lack of resources. Another reported suggestion is that the metabolism of proliferating cells also requires nutrients besides ATP for growth [15]. Further, a number of factors involved have been identified including protein kinase B (Akt), nuclear factor κB (NF-κB), hypoxia inducible factor 1 (HIF-1), and p53 [16]. It is believed that, in cancer cells, the pyruvate kinase PKM1 switches to the less active isoform PKM2. One of its functions is the conversion of phosphoenolpyruvate to pyruvate [17,18]. The loss-of-function mutations of succinate dehydrogenase and fumarate hydratase cause the accumulation of succinate and fumarate in mitochondria of cancer cells [17].

Increased conversion of glutamine (Gln) to glutamate (Glu) is another characteristic observed in cancer [17]. It has been suggested that proto-oncogene Myc binds to the promoter of the Gln importer and that this results in increased uptake of Gln [19]. Glutamine has multiple roles in the cell. It serves as an important source of nitrogen as well as carbon and is involved in signaling [20].

Beside this, redox homeostasis pathways are upregulated. An example can be serine metabolism which leads to the production of dihydronicotinamide-adenine dinucleotide phosphate (NADPH) and GSH [14]. Additionally, upregulated fatty acid synthase activity has been observed in cancer cells leading to elevated levels of lipids [21].

In this context, the knowledge of the influence of DiRu-1 on the metabolism of ovarian cancer cells will help us to elucidate its modes of action. For this purpose, the effect of DiRu-1 treatment on the metabolic changes in the A2780 and A2780cisR cell lines was investigated. The cells were treated with DiRu-1 in a dose corresponding to the IC_50_ of DiRu-1 (0.03 µM) and to a half-dose (0.015 µM). Non-tumor HEK-293 cell line was used to provide a reference.

## 2. Results

**Figure 2:**
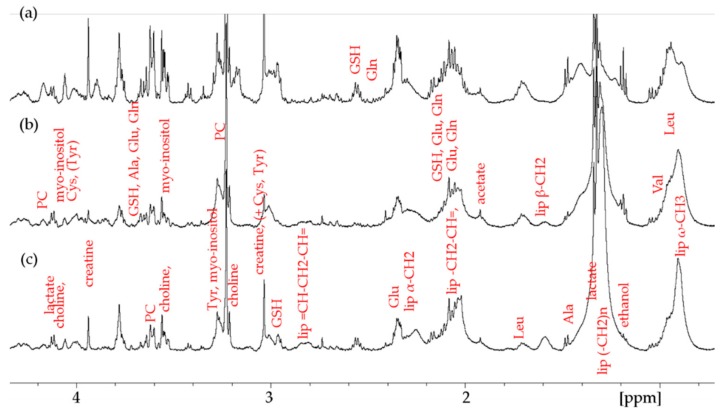
One-dimensional ^1^H NMR NOESY spectra of untreated (**a**) HEK-293; (**b**) A2780; and (**c**) A2780cisR cells: Each spectrum represents an average of 10 samples.

In this study, the cells were lysed prior to an NMR investigation in order to reach higher stability [22]. Therefore, the corresponding spectra obtained from cell lysates appeared somewhat different as compared to the whole cell spectra previously studied by our group on a hexacationic ruthenium metallaprism [23]. Overall, more individual metabolite peaks seem to be resolved in lysed cell spectra. Furthermore, there is a significant difference in observable signals such as the ones corresponding to the ω-CH_3_, (–CH_2_)_n_, and α-CH_2_ groups. Since these resonances are typical for the long alkyl chains present in various types of lipids such as phospholipids, fatty acids, or triglycerides, they will be referred to as lipid resonances throughout the manuscript. The ^1^H high resolution magic angle spinning (HR-MAS) NMR 1-D NOESY and T_2_-filtered PROJECT [23] spectra of the lysed and untreated cells are shown in Figure 2 and Figures S1 and S2, respectively.

In order to perform a statistical analysis, the spectra were manually divided into 52 buckets in the aliphatic and 44 buckets in the aromatic region (Tables S1 and S2). The bucketing was performed without prior knowledge of the individual metabolites present in order to avoid bias. First, 1-D ^1^H-NMR spectra recorded with the PROJECT sequence [23] were used for analyzing both aromatic and aliphatic regions. In these spectra, the T_2_-fitered suppression of broad components improves the visibility of small metabolite resonances as can be seen in Figure S1 compared to the spectra in Figure 2. In order to increase the reliability of the attribution of the lipid resonances, 1-D NOESY spectra were used (Figure 2) to specifically analyze the aliphatic region.

**Figure 3:**
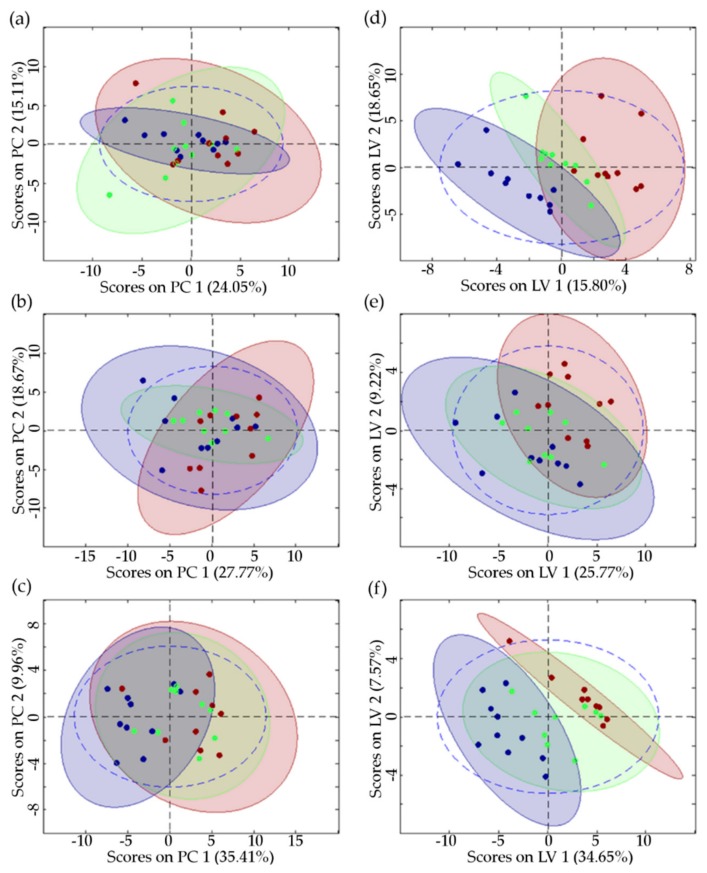
Principal component analysis (PCA; left side) and partial least squares method (PLS; right side) score plots of the aliphatic region (of 1-D PROJECT spectra) of (**a**,**d**) HEK-293, (**b**,**e**) A2780, and (**c**,**f**) A2780cisR cells treated with 0.03 μM of DiRu-1 (blue) or 0.015 μM of DiRu-1 (green) or untreated as the control (red). The ellipses show 95% confidence interval. The corresponding parameters are shown in Table 2.

The effects of DiRu-1 were statistically tested based on the bucketed spectra providing data matrices of 10 × 52 (aliphatic region) and 10 × 44 (aromatic region) for each group (10 replicates; control, treated with 0.015 µM, and treated with 0.03 µM) and each cell line. The models obtained from principal component analysis (PCA) showed some clustering of the three treatment groups but no separation between them (Figure 3 and Figure S3; Table 2). Partial least squares method (PLS, with the 3 treatment groups 0, 0.015, and 0.03 µM equally spaced as a y-table) showed complete separation between untreated and 0.03 µM of DiRu-1 treated A2780cisR groups. (Figure 3; Table 2). Partial least squares discriminant analysis (PLS-DA) for pair-wise comparison (controls versus 0.03 µM of DiRu-1 treatment) yielded good separation for all three cell lines, which was also the best for A2780cisR (Figure S4; Table S3). The corresponding PLS score plots applied to the aromatic regions are given in Figure S5.

Buckets that strongly contribute to the separation observable in the score plots of both PCA and PLS/PLS-DA (Figure 3 and Figures S3–S5) were identified using loading plots (Figures S6–S10). The first two components of each model served as a source for identification of contributing metabolites. Additionally, all other components contributing to the variance of the model by at least 10% were included as well. Next, we identified the metabolites present in these buckets using our own metabolite library, available metabolite databases [24,25] and data from the literature [26] using 2-D TOCSY spectra collected for each group of samples (Figures S11–S13).

**Figure 4:**
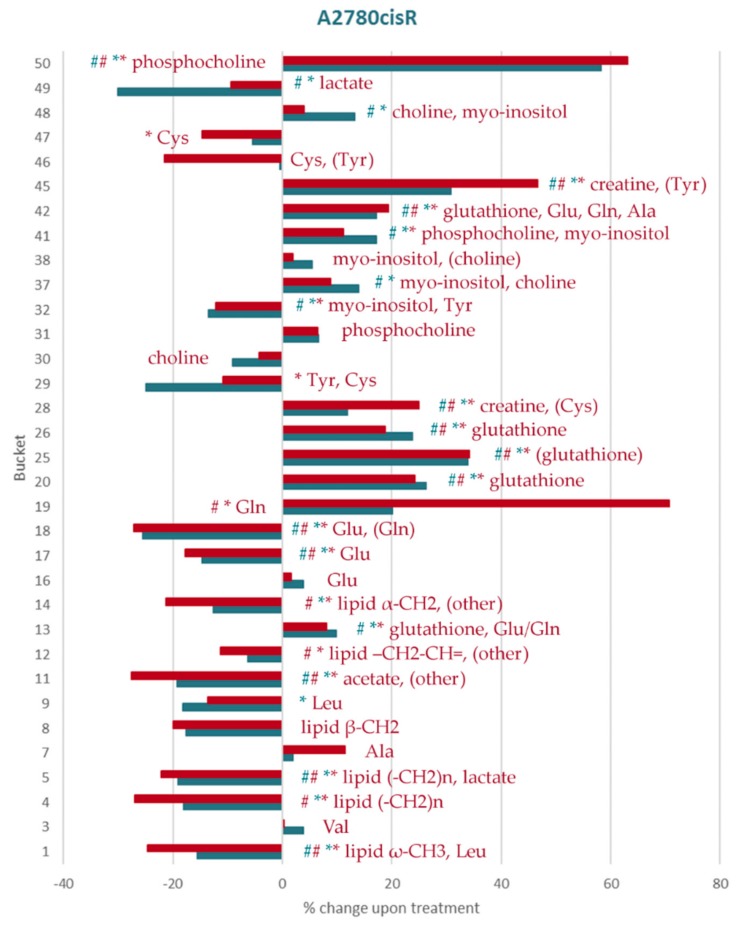
Percental change in the assigned aliphatic buckets in A2780cisR cells: The increasing/decreasing levels of each bucket compared to the control in cells treated with 0.015 µM of DiRu-1 (blue) and 0.03 µM of DiRu-1 (red) are expressed in percentages. The buckets marked with * show *p*-values < 0.05. The buckets marked with # show Benjamini–Hochberg-corrected *p*-values < 0.05. The complete bar plot also containing unidentified buckets is given in Figure S14.

**Figure 5:**
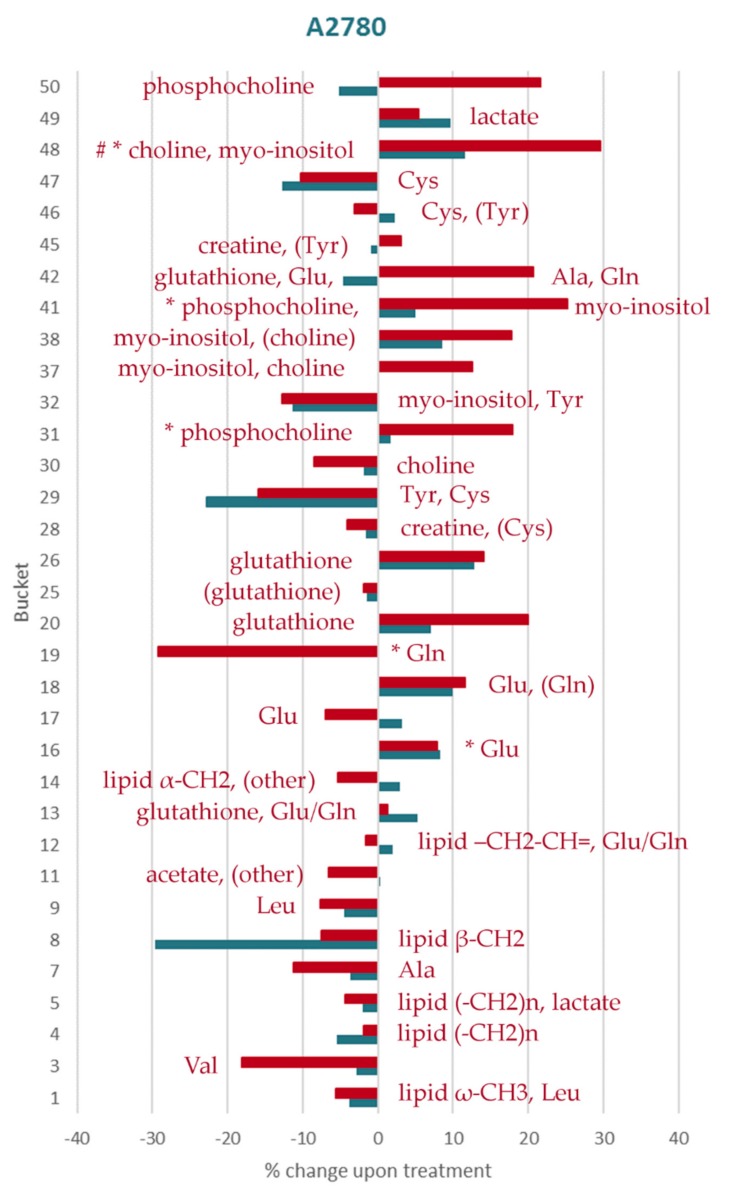
Percental change in the assigned aliphatic buckets in A2780 cells: The increasing/decreasing levels of each bucket compared to the control in cells treated with 0.015 µM of DiRu-1 (blue) and 0.03 µM of DiRu-1 (red) are expressed in percentages. The buckets marked with * show *p*-values < 0.05. The buckets marked with # show Benjamini–Hochberg-corrected *p*-values < 0.05. The complete bar plot also containing unidentified buckets is given in Figure S15.

**Figure 6:**
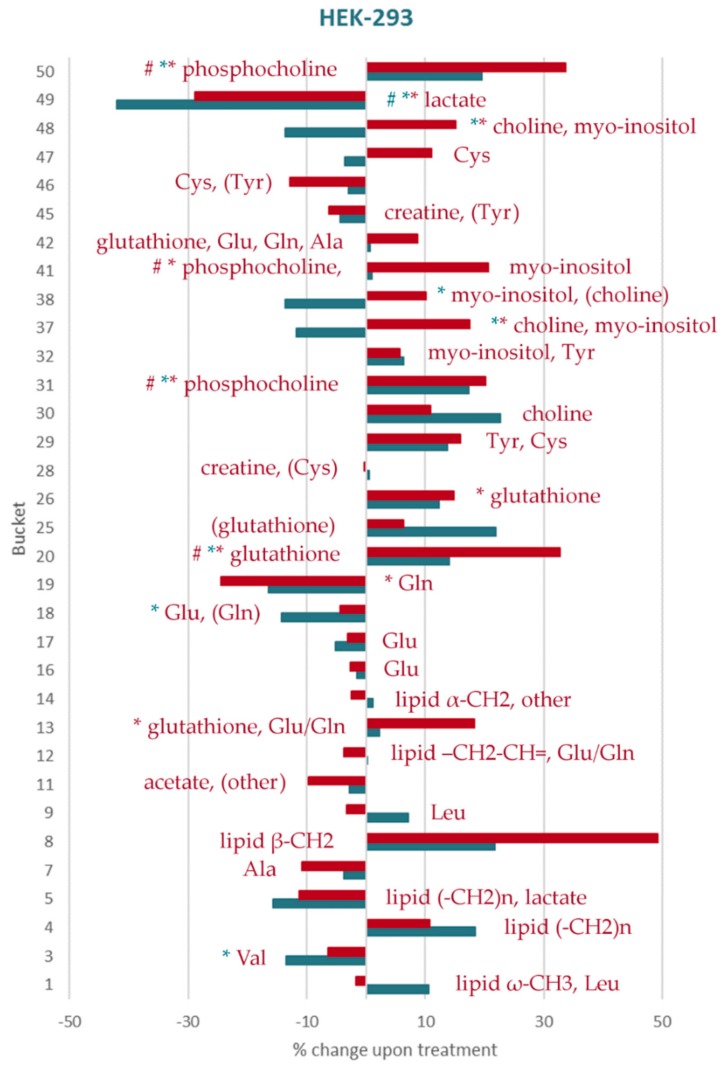
Percental change in the assigned aliphatic buckets in HEK-293 cells: The increasing/decreasing levels of each bucket compared to the control in cells treated with 0.015 µM of DiRu-1 (blue) and 0.03 µM of DiRu-1 (red) are expressed in percentages. The buckets marked with * show *p*-values < 0.05. The buckets marked with # show Benjamini–Hochberg-corrected *p*-values < 0.05. The complete bar plot also containing unidentified buckets is given in Figure S16.

**Figure 7:**
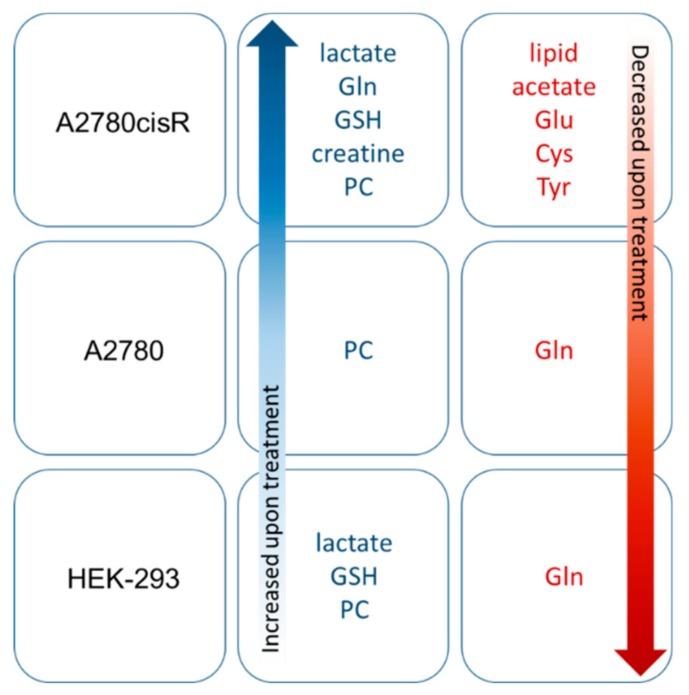
Summary showing the most significant changes in identified metabolites observed in the three cell lines upon treatment with DiRu-1: For a complete overview including the influence of the dose see Figure 4, Figure 5 and Figure 6, Figures S14–S25 and Table 3.

Identified metabolites and changes in their levels are summarized in Table 3. The percentage changes of the individual aliphatic buckets content upon treatment with both doses of DiRu-1 in relation to untreated cells has been summarized for the three different cell lines in Figure 4, Figure 5 and Figure 6 and Figures S14–S16 and for the aromatic buckets in Figures S17–S19. A comparison of bucket means can be found in Figures S20–S25. The treatment with a DiRu-1 dose of 0.03 µM led to the highest number of significant bucket changes. Interestingly, most of the significant changes were observed for the A2780cisR cell line (Figure 4 and Figures S14, S17, S20, S23), while the least number of significant changes was observed for the A2780 cell line (Figure 5 and Figures S15, S18, S21, S24). HEK-293-induced changes were lying in the middle (Figure 6 and Figures S16, S19, S22, S25). For the sake of clarity, a simplified overview of the most important results is provided in Figure 7.

### 2.1. Lipid Metabolism

The levels of lipids based on the evaluation of individual detected groups were significantly altered only for the A2780cisR cell line. Decreases were observed after DiRu-1 treatment for the groups that were clearly identified, namely ω-CH_3_, (–CH_2_)_n_, lipid β-CH_2_, and α-CH_2_, and were significant for ω-CH_3_, (–CH_2_)_n_, and α-CH_2_. Of note, the decreases were also significant for ω-CH_3_, (–CH_2_)_n_, and α-CH_2_ when half of the IC_50_ dose was applied.

The phosphocholine level increased significantly in both treated A2780cisR and HEK-293 cell lines, and the choline level did not change significantly upon treatment with DiRu-1 in any of the tested cell lines.

### 2.2. Amino Acid and GSH Metabolism

GSH was upregulated especially in A2780cisR and to a smaller extent also in the HEK-293 cell line. Using the lower dose of 0.015 µM, the significant upregulation occurred only in A2780cisR cells.

The glutamine (Gln) level changed in all three cell lines but only upon treatment with 0.03 µM of DiRu-1. Interestingly, a significant increase of the Gln level was observed for A2780cisR while a decrease was observed for the other two cell lines. On the other hand, the glutamate (Glu) level decreased significantly in A2780cisR already with a 0.015 µM dose of DiRu-1.

Amino acids cysteine (Cys), alanine (Ala), leucine (Leu), tyrosine (Tyr), and phenylalanine (Phe) showed a trend towards increased or decreased values, which, however, were not significant after correcting for multiple comparisons. All of the changes to be considered occurred in the A2780cisR cell line with the exception of Phe which decreased consistently only in A2780.

### 2.3. Sugar-Containing Compounds

The myo-inositol showed an increase in one of the two buckets in A2780cisR when the 0.015 µM dose of DiRu-1 was applied. Overall, most of the changes on myo-inositol were not significant across the cell lines.

The changes in AMP were not very consistent in any of the cell lines. The AMP level decreased in one of the two buckets in the case of the A2780 cell line treated with DiRu-1. There were no consistent changes in the other two cell lines.

The uracil level changed consistently only in A2780cisR where it showed an increase upon DiRu-1 treatment but was not significant. In the other two cell lines, the changes were neither consistent nor significant. In general, changes in uracil, uridine diphosphate (UDP), and uridine triphosphate (UTP) levels were not very consistent, as can be seen in Table 3. It was not possible to distinguish sufficiently cytidine (Cyt) and uridine (Urd) so that these metabolites had to be analyzed together.

### 2.4. Other Metabolites

The creatine level increased significantly in A2780cisR treated with DiRu-1. The change was significant for both doses and in both buckets assigned to creatine (Cre). No consistent Cre changes were observed in the A2780 or HEK-293 cell lines treated with the complex.

The lactate level dropped significantly in the A2780cisR and HEK-293 cell lines upon treatment with DiRu-1. In both cases, the effect was significant when using the 0.015 –µM DiRu-1 dose.

The acetate level decreased significantly only in A2780cisR cells when treated with any of the two doses of DiRu-1.

Further, there were yet unidentified buckets 6, 10, 15, 21, 23, 27, 35, and 43 which showed significant changes in A2780cisR. Bucket 10 showed over 100% increase when treated with any of the two doses compared to the control. A somewhat smaller but still very high increase was observed also for bucket 35. Buckets 21, 23, 35, and 39 showed significant changes in the HEK-293 cell line as well.

### 2.5. Correlation Analysis

**Figure 8:**
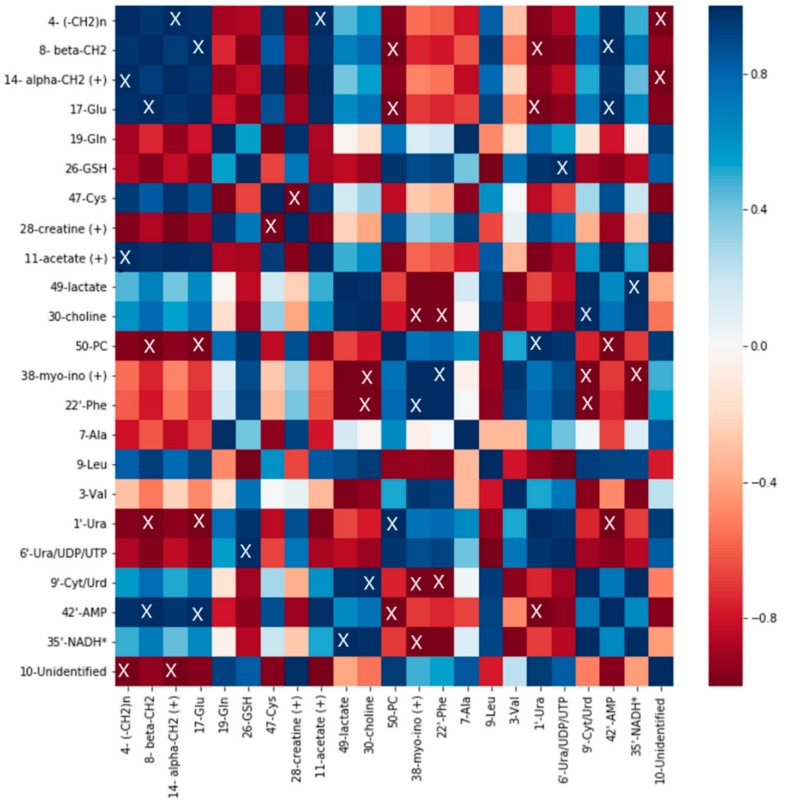
Heatmap of A2780cisR cells treated with 0.03 µM of DiRu-1 vs. control: The blue color scale has been used for positive correlation, and the red scale is for negative correlation. “X” indicates significances with *p*-values < 0.05. * Tentative assignment. (+) stands for the possibility that some other metabolite is contained in the bucket in question in addition to the assigned metabolite.

**Figure 9:**
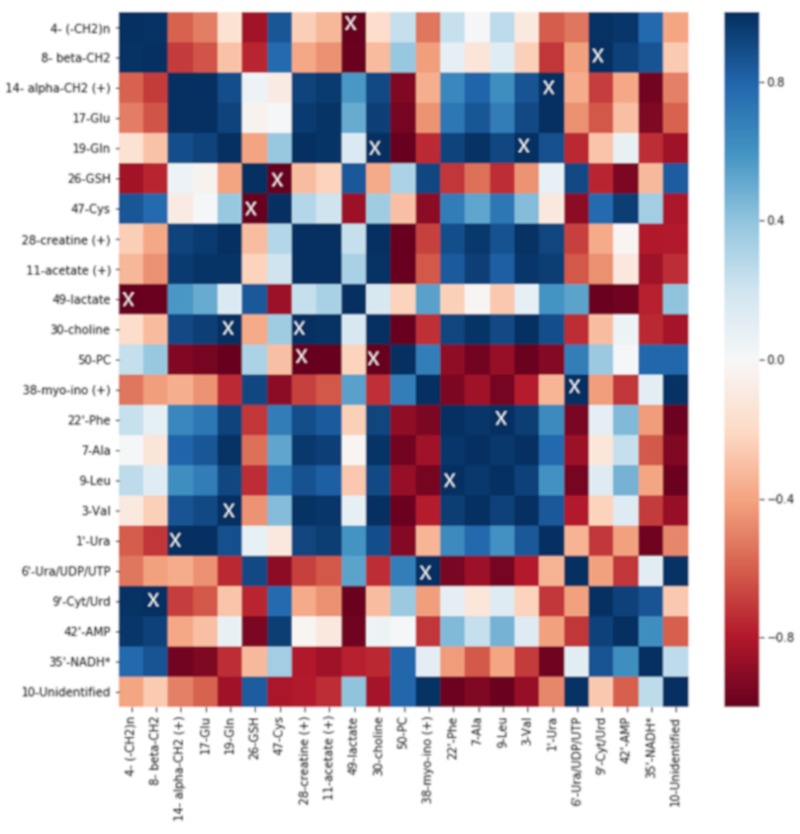
Heatmap of A2780 cells treated with 0.03 µM of DiRu-1 vs. control: The blue color scale has been used for positive correlation, and the red scale is for negative correlation. “X” indicates significances with *p*-values < 0.05. * Tentative assignment. (+) stands for the possibility that some other metabolite is contained in the bucket in question in addition to the assigned metabolite.

**Figure 10:**
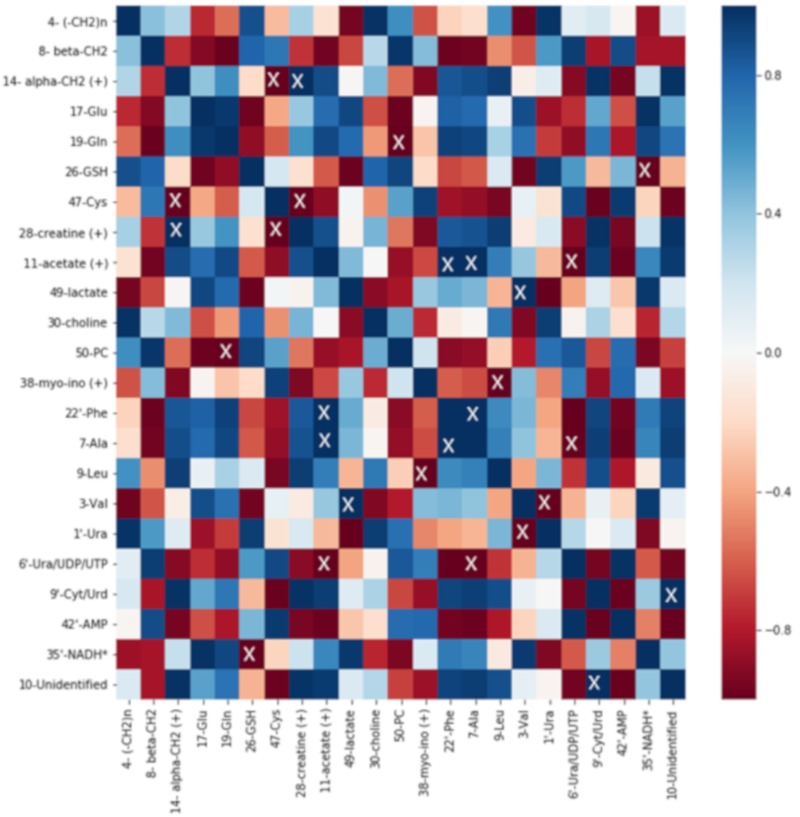
Heatmap of HEK-293 cells treated with 0.03 µM of DiRu-1 vs. control: The blue color scale has been used for positive correlation, and the red scale is for negative correlation. “X” indicates significances with *p*-values < 0.05. * Tentative assignment. (+) stands for the possibility that some other metabolite is contained in the bucket in question in addition to the assigned metabolite.

Correlation analysis was performed on normalized integral averages using the Pearson algorithm. The results are visualized as heatmaps. For every cell line, control and 0.03 µM DiRu-1 treated groups were tested. For each identified metabolite, a representative bucket was chosen (Figure 8, Figure 9 and Figure 10).

The heatmaps give a complex overview over the correlations among a number of different metabolites identified in the cell spectra. From the heatmaps, it is observed that the correlation of Glu with Gln is, to some extent, positive in the A2780 and HEK-293 cell lines in contrast to the A2780cisR cell line where negative correlation can be observed. GSH is positively correlated with Ura/UDP/UTP in all three cell lines and in A2780cisR significantly. It is further negatively correlated with a bucket assigned to nicotinamide adenine dinucleotide (NADH) in all three cell lines and in HEK-293 significantly. Lipid levels based on β-CH_2_ and (–CH_2_)_n_ seem to be correlated positively with acetate level as well as with Glu and AMP and negatively with phosphocholine and Ura in the A2780cisR cell line. In the A2780 and HEK-293 cell lines, the correlations were not uniform across different lipid signals.

## 3. Discussion

Over the last years, our group developed a number of trithiolato bridged diruthenium *p*-cymene complexes. The most active compound DiRu-1 showed significant cytotoxicity against different cancer cell lines. Nevertheless, its mode of action is still not fully understood. It has been shown that it is able to catalyze GSH oxidation, but this mechanism can only partly account for the measured cytotoxicity. Interestingly, recent ICP-MS studies showed that up to 97% of Ru is localized in the mitochondria of treated cancer cells [11].

In this study, three cell lines were treated with DiRu-1 and analyzed using ^1^H-HR-MAS NMR spectroscopy. We show that the treatment of the cells with DiRu-1 at a dose of 0.03 µM (IC_50_) induces significant changes in the metabolic profile of the A2780cisR cell line and to a less extent of the metabolic profile of the HEK-293 cell line. Interestingly, the metabolic profile of the A2780 cell line seems to be less affected upon treatment with DiRu-1, suggesting a different mode of action in the two A2780 cell lines.

In A2780cisR cells, we have observed that (i) the GSH level increased significantly even after treatment with the 0.015 µM dose of DiRu-1 and that (ii) the Gln level increased and (iii) the Glu level dropped using the 0.03 µM dose. The Cys level drop with lesser significance was additionally observed. The increase in GSH levels was observed to some extent in the other two cell lines as well but with less significance. Similarly, an increase in GSH in both cancer cell lines has been observed in cells treated with a Ru prism [26]. Elevated GSH levels can be commonly found in cells under oxidative stress. Since reactive oxygen species (ROS) production is increased in cancer cells, it is followed by an increase in levels of antioxidants such as GSH in response. More than 10% of the GSH synthesized within the cell is located in the mitochondria, preventing apoptosis [27]. Since DiRu-1 is able to catalyze GSH oxidation, it is likely that it leads to further increase in oxidized glutathione (GSSG) levels. In this context, it would have been helpful if the ratio of GSSG and GSH could have been determined. While GSH was identified, GSSG could not be clearly distinguished to provide this information. Similar observations have been reported by Duarte et al. in 2010 [28].

Gln level increased while Glu showed a significant drop in the A2780cisR cell line while no such consistent changes were observed in the other two cell lines. A negative correlation, yet not statistically significant, has been observed in the heatmap for A2780cisR cells. On the contrary, the correlation in the other two cell lines was positive for these two metabolites. Gln has multiple roles in cells. It serves as a source of carbon; reduces nitrogen; and is involved in the production of GSH, nucleotides, and lipids. Further, it seems to be involved in cell signaling. In cancer, it has multiple roles including neoplasm manifestation, tumor progression, and metastasis. Gln metabolism varies with organs, and different subtypes of cancer can show distinct metabolic patterns. Glu, the first product of Gln catabolism, is either consumed or released from the cell. The efflux is coupled to Cys influx [29,30,31]. Both Gln and Glu were found to be increased in ovarian cancer cysts, tissue, and serum as reported in the literature [32]. The increase in the Gln level accompanied with a drop in the Glu level found here may indicate decreased glutaminolysis.

Cys availability is one of key factors in the first step of GSH synthesis [33]. As reported by Nunes et al., Cys, which has a protective role in ovarian cancer cells under hypoxic conditions, seems to be involved in the chemoresistance of A2780cisR cells [34]. We observed decreased levels of Cys in A2780cisR cells treated with DiRu-1, which may indicate the ability of DiRu-1 to overcome the A2780cisR resistance, as highlighted by the identical IC_50_ values against both A2780 cell lines. Also, Cramer et al. showed that the depletion of Cys following the introduction of a pharmacologically optimized cysteinase resulted in cell death of cancer cells due to the depletion of GSH and the accumulation of ROS [35]. The decrease in the Cys level can be related to increased levels of GSH.

The level of creatine increased significantly only in A2780cisR cells; this effect occurred even when the lower of the doses was applied. Creatine plays a number of roles in a cell. Among other functions, it is an antioxidant with possible protective activity on oxidatively damaged mitochondria [36,37,38]. It has been suggested to have an anticancer effect. Decreased creatine and creatine kinase levels, on the other hand, have been reported in the development of sarcoma. Low serum creatine kinase has been also observed in breast cancer patients [38,39,40]. The increased creatine level observed in A2780cisR cells could have similar reasons as in the case of GSH. The presence of a Ru complex could lead to increased production of creatine, which in turn could contribute to the protection of mitochondria from ROS.

Lipids, at least as represented by the ω-CH_3_, (–CH_2_)_n_, lipid β-CH_2_, and α-CH_2_ groups, are another group of metabolites that changed significantly only in A2780cisR cells. A similar effect, a decrease in the detected level of lipids, has been earlier described for A2780 cells treated with a Ru prism. The same study reported an increase in detected lipid levels in treated HEK-293 cells and inconsistent changes in A2780cisR cells [26]. Herein, we report a significant decrease in detected lipid levels observed, specifically in the A2780cisR cell line. Cancer cells are known to have an altered lipid metabolism. Elevated levels of carnitine and its derivatives were reported in patients with ovarian cancer, and the long chain fatty acids content was found to be increased in serum and tissue in a number of studies [16,32]. The decrease of the detected lipid content in A2780cisR cells treated with DiRu-1 may be related to a downregulation of their biosynthesis, decreased uptake, hindered distribution, or storage in form of droplets, as shown by others [21,41,42,43].

The lactate level decreased significantly in both the A2780cisR and HEK-293 cell lines. A similar effect has been earlier observed for a Ru prism [26]. Higher lactate levels are associated with carcinogenesis as part of the Warburg effect, and lactate production has even been suggested to be its purpose. Increased levels of lactate and lactate dehydrogenase in serum have been reported to be highly associated with poor prognoses [44,45,46]. A higher content of lactate was reported also specifically for ovarian cancer cyst fluids and primary and metastatic tissue in a number of studies [32]. Lowering lactate levels upon DiRu-1 action could possibly be, therefore, a good sign. To get more insight into the observed changes, it might be suitable to perform measurements of extracellular and intracellular lactate levels which would be useful also for other metabolites but was beyond the scope of this study.

When evaluating the results of this study, certain limitations needs to be taken into account. Analogously to the earlier study reported by our group [26], we chose the HEK-293 cell line as a reference cell line. Limitations of HEK-293 need to be considered when comparing the results with A2780 and A2780cisR as it does not have a stable karyotype and shows traits common with cancer cells such as overexpression of certain genes [47]. Further, it is grown in a DMEM medium compared to A2780 and A2780cisR, which are grown in RPMI-1640. As far as metabolite quantification is concerned, there is a common problem with overlap of individual metabolite peaks which may lead to loss of useful information. Fitting of single resonances would be an approach to address this problem; on the other hand, it may lead to the disregard of unknown components. In this study, we applied correlation analyses combined with significance tests of single buckets to support the finding of the PCA and PLS analyses. Manual bucketing was applied prior to the assignment of metabolites in order to reduce bias. Further options would be bucketing according to metabolite assignment and automated or semi-automated assignment and quantification. Finally, it needs to be taken into account that the results we report here derive from an in vitro study and may therefore differ from an in vivo study.

Most of the significant changes were observed in A2780cisR cells, and while the lower dose of DiRu-1 (IC_50_/2) shows yet a number of significant changes, it appears that IC_50_ represents the necessary dose to observe the most significant changes. Interestingly, there are significant differences in the metabolic responses of A2780 and A2780cisR cells treated with DiRu-1 despite responding to the same IC_50_ value of DiRu-1 as has been shown previously. It is possible that the mechanism of DiRu-1 action differs in the two cell lines. The exact reasons behind this observation remain yet to be explained.

## 4. Materials and Methods

### 4.1. Synthesis

DiRu-1 was prepared according to formerly published procedures [10].

### 4.2. Cell Culture and Treatment

Human ovarian carcinoma cells A2780 and the Cis-Pt resistant variant cells A2780cisR were obtained from the European Center of Cell Cultures (ECACC, Salisbury, UK). The HEK-293 cell line was provided by Prof. Mühlemann, University of Bern.

A2780 cells were grown in Roswell Park Memorial Institute (RPMI) 1640 medium containing 10% fetal calf serum (FCS), 2 mM Gln, and 1% antibiotics (penicillin/streptomycin) at 37 °C and 5% CO_2_. HEK-293 cells were grown in Dulbecco’s Modified Eagle Medium (DMEM) with the same supplements as in the previous case with additional 1% HEPES pH buffer.

Cytotoxicity tests of DiRu-1 for A2780 and A2780cisR were determined previously using the 3-(4,5-dimethylthiazol-2-yl)-2,5-diphenyltetrazolium bromide (MTT) assay [10]. In this context, it should be also mentioned that cytotoxicity tests performed on other trithiolato bridged diruthenium complexes closely related to DiRu-1 towards HEK-293 exhibited very similar IC_50_ values when compared to ovarian cancer cells A2780, indicating no high selectivity [26,48,49].

In each group, 10 independently prepared samples have been measured, leading to a total number of 90 measurements (10 × 3 × 3; 3 cell lines, 3 groups per cell line: control, low dose, and high dose). The cells used for the metabolomics study were firstly seeded in 25 cm^2^ flasks with a density of 8 × 10^4^ cells/cm^2^ and left to grow for 24 h at 37 °C in a humidified incubator in the presence of 5% CO_2_. The incubation time of 24 h has been chosen as in our previous study on a similar Ru complex; we have shown that 24 h incubation was adequate to observe a sufficient effect [26].

Afterwards, the medium in which the cells were grown was disposed. Subsequently, the cells were washed with 5 mL of phosphate-buffered saline (PBS). For drug treatment, 5 mL of fresh DMEM or RPMI-1640 medium were added to the flasks containing 3 µL aqueous solutions of DiRu-1 to reach a final concentration of 0.03 µM or 0.015 µM, respectively, or solely H_2_O (controls).

After 24 h of incubation, the medium was disposed and cells were washed with 5 mL of PBS. Subsequently, trypsin/ethylenediaminetetraacetic acid (EDTA) was added, and after ~5 min, when the cells detached, 1 mL of DMEM was added and cells were transferred into new Falcon tubes. Cell viability was determined using 10 µL of 0.4% Trypan Blue Stain for 10 µL of cell suspension. An automatic cell counter was used for this purpose. The cells were further centrifuged and, after disposal of the supernatant, resuspended in freezing medium (10 mL of FBS, 9 mL of DMEM, and 1 mL of DMSO). The cell suspension was then transferred into cryovials and kept frozen at −80°C.

To prepare the cell lysate samples for NMR analysis, the cells were thawed at 37 °C, transferred into 1 mL of preheated medium, and washed three times with PBS. The pellet was resuspended in 20 µL of PBS-D_2_O, alternatingly sonicated for 30 s and dipped into liquid nitrogen for 15 s three times, and finally frozen in liquid N_2_ and stored at −80 °C.

### 4.3. HR-MAS NMR

Each sample was thawed at 37 °C directly before measurements. The cell lysate was pipetted into a 4-mm MAS rotor, and the volume was regulated with a 12-µL insert. The closed rotor was inserted into a dual inverse ^1^H^13^C HR-MAS probe-head of a 500 MHz spectrometer (Avance II, Bruker BioSpin, Fällanden, Switzerland) and left to equilibrate with the temperature set to 272 K while being spun at 5 kHz at the magic angle. Two types of one-dimensional ^1^H NMR spectra both with presaturation of the water resonance have been recorded for each sample: a 1-D NOE spectrum, using the pulseprogram *noesypr1d* taken from the Bruker pulse sequence library, and a 1-D PROJECT [23] spectrum, applying a T_2_-filter of 102.4 ms for suppression of broad components. The spectra were recorded with a spectral width of 6002 Hz (12 ppm), 32,768 data points, 4 s relaxation delay, and 512 scans for NOESY and 1024 scans for PROJECT, respectively. Additionally, 2-D ^1^H-^1^H TOCSY experiments (pulseprogram *dipsi2phpr* from the Bruker pulse sequence library) and 2-D ^1^H *J*-resolved experiments (pulseprogram *jresgpprqf* from the Bruker pulse sequence library) were recorded for two samples of each group to help with the assignment of individual metabolites.

All spectra have been identically processed using an exponential multiplication with a line broadening of 1 Hz. All spectra have been calibrated with respect to the phosphocholine resonance at 3.23 ppm. The baseline correction has been performed independently for two regions: the upfield (0.350–4.793 ppm) part and the downfield (5.575–9.001 ppm) part for avoiding the residual water resonance. The processed spectra have been exported as *ascii* files that served as an input for statistics.

### 4.4. Statistics and Data Analysis

The ascii files have been divided into groups (cell line and dose). The aliphatic and aromatic regions were treated separately. The regions of spectra were divided into buckets, each bucket representing resonances of individual metabolites wherever possible. Fifty-two buckets were defined in the aliphatic region in NOESY spectra. Forty-four buckets were defined for the aromatic region of the PROJECT spectra.

Probabilistic quotient normalization (PQN) was applied to all integrals in the analyzed set. The type of scaling used and further details on the analysis can be found in summary in Table 2 and Table S10.

Division of the data into groups according to cell line and DiRu-1 dose enabled evaluation of the effect of DiRu-1 and dose within one cell line, general differences in metabolism of three different cell lines, and comparison of the treatment effect in different cell lines. The preprocessed data were then used as an input for principal component analysis (PCA) of the groups. The highest contributing principal components (PC-1-PC-3) have been plotted in PCA score plots. Outliers including cases where the quality of the sample was insufficient and/or low S/N spectra have been excluded from the analysis (Table 2 and Table S10).

Next, partial least squares (PLS) and/or partial least squares discriminant analysis (PLS-DA) were performed using the PLS toolbox software (Eigenvector Research, Inc., Manson WA, USA). Similar as in the case of PCA, the latent variables for PLS or PLS-DA that contributed the most to the models have been plotted.

Venetian blinds method was used for cross-validation. Further details on statistical analysis are summarized in Table 2 and Table S10.

A correlation matrix was calculated in each cell line for the control and 0.03 µM DiRu-1 groups using the Pearson algorithm. Subsequently, corresponding heatmaps were produced.

The buckets contributing to a variance in the first two components of the PCA, PLS, or PLS-DA models above an arbitrary threshold loading value of 0.2 were assigned—where possible—to metabolites. Additionally, buckets above the same loading value of other components contributing more than 10% to the variance were considered. The metabolites were identified using 1-D ^1^H NOESY, PROJECT, and 2-D TOCSY spectra (Figures S11–S13). As reference metabolite databases, the Biological Magnetic Resonance Data Bank (BMRB) and the Human Metabolome Database (HMDB) [50] were used in addition to the internal metabolite library. Significance testing was performed using the t-test, and corrections for multiple comparisons were performed using the Benjamini–Hochberg method [51]. For the buckets in question, a change upon treatment was considered significant for *p*-values < 0.05.

The following list of software programs was used: Topspin 3.5pl7 (Bruker Biospin, Billerica, MA, USA); Matlab R2012a (Mathworks, Natick, MA, USA) and PLS Toolbox (Eigenvctor Research Inc., Manson, WA, USA) for Matlab; Python 3.7 (Python Software Foundation, Wilmington, DE, USA). The data were stored using MetaboLights data repository [52].

## 5. Conclusions

In this study, we treated three cell lines with DiRu-1 and analyzed their lysed suspensions using HR-MAS NMR spectroscopy. The treatment showed significant metabolic changes mainly in the A2780cisR cell line. The A2780 cell line exhibited changes, which, however, were not significant. It turned out that the full IC50 dose of 0.03 µM of DiRu-1 was required to observe the treatment effect at a sufficient significance level.

The levels of GSH and creatine increased in the A2780cisR cell line upon treatment with DiRu-1. The increase in GSH and creatine levels can be related to their function as antioxidants. Changes affecting redox homeostasis can be related to the fact that DiRu-1 is able to catalyze GSH oxidation as it has been shown earlier. Furthermore, the Gln level was increased in A2780cisR cells treated with DiRu-1 while the Glu level decreased. The combination of increased Gln and decreased Glu could mean reduced glutaminolysis.

Another group of metabolites affected were lipids. The overall decrease in detected lipid levels in A2780cisR cells undergoing treatment with DiRu-1 could be explained by downregulated biosynthesis, decreased uptake, or a lack of lipid storage and distribution.

Last but not least, lactate levels have been observed dropping in A2780cisR cells treated with DiRu-1. As high lactate has been reported to be a sign of poor prognosis for a cancer patient, we consider a decrease in lactate a sign of positive drug response.

It can be concluded that DiRu-1 caused most metabolic changes in the A2780cisR cell line and that the changes observed are partially related to the specific DiRu-1 structure and its related nature.

## Figures and Tables

**Table 1:** IC_50_ (µM) of DiRu-1 against various cancer cell lines [9,10].

Cell Line	A2780	A2780cisR	MCF-7	MDA-MB-231	HepG2
IC_50_	0.03	0.03	0.077	0.228	0.268

**Table 2:** Summary of parameters and statistical results of the PCAs and PLS shown in Figure 3. * In the case of PLS for both the x and y tables ** Values below 0.05 indicate significance at the 95% level.

Groups	HEK-293	A2780	A2780cisR
Included samples	(1–30) (1–52)	(1–17 19-30) (1–52)	(1–19 21–23 25-30) (1–52)
Preprocessing *	mean center, UVS		
Cross validation	Venetian blinds 5 splits, 1 sample/split		
X-block	30 × 52	29 × 52	28 × 52
**PCA**			
Components	4	2	2
Algorithm	SVD	SVD	SVD
RMSEC	0.599	0.719	0.726
RMSECV	0.969	0.962	0.976
Total variance captured	62.94%	46.45%	45.37%
PC 1	24.05%	27.77%	35.41%
PC2	15.11%	18.67%	9.96%
PC3	12.60%	-	-
PC4	11.18%	-	-
**PLS**			
Number of LVs	2	4	2
Algorithm	SIMPLS	SIMPLS	SIMPLS
RMSEC	0.354	0.337	0.385
RMSECV	0.533	0.904	0.571
R^2^Cal	0.812	0.835	0.781
R^2^CV	0.576	0.096	0.524
Wilcoxon (self-pred.) **	0.010	0.113	0.069
Wilcoxon (cross-val.) **	0.004	0.085	0.014
Sign test (self-pred.) **	0.068	0.254	0.197
Sign test (cross-val.) **	0.031	0.140	0.067
Rand t-test (self-pred.) **	0.014	0.194	0.066
Rand t-test (cross-val.) **	0.006	0.190	0.016
Total variance captured	34.45%	54.83%	42.22%
LV 1	15.80%	25.77%	34.65%
LV 2	18.65%	9.22%	7.57%
LV 3	-	14.28%	-
LV 4	-	5.56%	-

**Table 3:** Up- or downregulated metabolites upon treatment with DiRu-1. * indicates that the changes observed were significant with a *p*-value < 0.05. The buckets marked with # show Benjamini–Hochberg-corrected *p*-values < 0.05. Abbreviations: ar = buckets in aromatic region; Ac = acetate; Ala = alanine; AMP = adenosine monophosphate; Chol = choline; Cre = creatine; Cyd = cytidine; Cys = cysteine; Fum = fumarate; Glu = glutamate; Gln = glutamine; GSH = glutathione; Lac = lactate; Leu = leucine; myo-Ino = myo-inositol; NADH = Nicotinamide adenine dinucleotide; PC = phosphocholine; Phe = phenylalanine; Tyr = tyrosine, UDP = uridine diphosphate; Ura = uracil; Urd = uridine; and UTP = uridine triphosphate. ? means assigned with not high enough certainty.

	HEK-293		A2780		A2780cisR	
	DiRu-1 (µM)		DiRu-1 (µM)		DiRu-1 (µM)	
**Metabolite (bucket)**	**0.015**	**0.03**	**0.015**	**0.03**	**0.015**	**0.03**
lipid ω-CH_3_ (+Leu) (1)	0	↑	↓	↓	↓ * #	↓ * #
lipid (–CH_2_)_n_ (4)	↑	↑	↓	↓	↓ *	↓ * #
lipid (–CH_2_)_n_, Lac (5)	↓	↓	↓	↓	↓ * #	↓ * #
lipid β-CH_2_ (8)	↑	↑	↓	↓	↓	↓
lipid –CH_2_–CH=, Glu/Gln (12)	0	0	0	↓	↓	↓ * #
lipid α-CH_2_ + other (14)	0	0	0	↓	↓ *	↓ * #
Lac, lipid (–CH_2_)_n_ (5)	↓	↓	↓	↓	↓ * #	↓ * #
Lac (49)	↓ * #	↓ *	↑	↑	↓ * #	↓
Ala (7)	↓	↓	↓	↓	↑	↑
Leu (9)	↑	↓	↓	↓	↓	↓ *
Ac (+other) (11)	↓	↓	0	↓	↓ * #	↓ * #
Glu/Gln, lipid –CH_2_–CH= (12)	0	0	x	x	↓	↓ * #
Glu (16)	0	0	↑ *	↑	0	0
Glu (17)	↓	0	↑	↓	↓ * #	↓ * #
Glu, Gln (18)	↓ *	↓	↑	↑	↓ * #	↓ * #
Gln (19)	↓	↓ *	0	↓ *	↑	↑ * #
GSH, Glu, Gln (13)	0	↑ *	0	↑	↑ * #	↑ *
GSH (20)	↑ *	↑ * #	↑	↑	↑ * #	↑ * #
(GSH) (25)	↑	↑	0	0	↑ * #	↑ * #
GSH (26)	↑	↑ *	↑	↑	↑ * #	↑ * #
Cre (+Cys?) (28)	0	0	0	↓	↑ * #	↑ * #
Cre (+Tyr) (45)	0	↓	0	0	↑ * #	↑ * #
Cys, Tyr (29)	↑	↑	↓	↓	↓	↓ *
Cys (+Tyr) (46)	↓	↓	0	0	0	↓
Cys (47)	0	↑	↓	↓	↓	↓ *
Tyr, Cys (29)	↑	↑	↓	↓	↓	↓ *
Tyr, myo-Ino (32)	↑	↑	↓	↓	↓	↓ * #
(Tyr), Cys (46)	0	↓	0	0	0	↓
Chol (30)	↑	↑	0	↓	↓	0
PC (31)	↑ *	↑ * #	0	↑ *	↑	↑
Chol, myo-Ino (37)	↓	↑ *	0	↑	↑ * #	↑
(Chol), myo-Ino (38)	↓ * #	↑	↑	↑	↑	↑
Chol, myo-Ino (48)	↓ *	↑ *	↑	↑ * #	↑ * #	↑
myo-Ino, Tyr (32)	↑	↑	↓	↓	↓ * #	↓ *
myo-Ino, Chol (37)	↓	↑ *	0	↑	↑ * #	↑
myo-ino, PC (41)	0	↑ * #	0	↑ *	↑ * #	↑ *
GSH, Glu, Gln, Ala (42)	0	↑	↓	↑	↑ * #	↑ * #
PC (50)	↑ *	↑ * #	↓	↑	↑ * #	↑ * #
Ura (1 ar)	↑	↑	↑	↓	↑	↑
Ura (23 ar)	↓	↓	↑	↓	↑	↑
Ura (24 ar)	0	↓	↑	0	↑	↑
Ura/UDP/UTP (5 ar)	↑	↑	↑	↑	↑	↑
Ura/UDP/UTP (6 ar)	0	↑	↑	↑ *	↑	↑
Ura/UDP/UTP (7 ar)	↓	0	0	↑ *	↑	↑
Ura/UDP/UTP (8 ar)	↓	↓	↓	↑	0	↓
Cyd/Urd (3 ar)	↑	↑	0	↓	↓	0
Cyd/Urd (9 ar)	0	↓	↓	0	↓ *	↓
Tyr (14 ar)	0	0	0	↑	↓	↓
Tyr (15 ar)	↑	↑	0	↓	↓	↓ *
Tyr (18 ar)	0	↓	↓	↓	↓ *	↓
Phe (20 ar)	↑	0	↓	↓	0	↓
Phe (22 ar)	↓	↓	↓	↓ *	↑	↑
UDP (32 ar)	↑	↑	↑	↑	↑	↓
UDP/UTP (31 ar)	0	↑	↑	↑	↑	↑
AMP (37 ar)	↓	↑	0	0	0	↑
AMP (42 ar)	0	↑	↓	↓ *	0	0
Fum? (13 ar)	0	↑	0	↑	↑	0
NADH? (12 ar)	↓	↑	0	↑	↓	0
NADH? (35 ar)	↓	↓	↓	↑	↓ *	↓
NADH? (36 ar)	↓	0	↓	↓	↓	↓
NADH? (40 ar)	0	↑	0	↑	↑	↑

## Supplementary Materials

The following are available online at https://www.mdpi.com/2218-1989/9/7/146/s1, Figure S1: One-dimensional 1H NMR PROJECT spectra of the three untreated cell lines; Figure S2: Aromatic region of PROJECT spectra of the three untreated cells lines; Table S1: The 52 buckets in the aliphatic region of the 1-D 1H NMR spectra (0.79–4.40 ppm) and the corresponding metabolites; Table S2: The 44 buckets in the aromatic region of the 1-D 1H NMR spectra (5.78–8.95 ppm) and the corresponding metabolites; Figure S3: PCA score plots of the aromatic region of the three cell lines treated with DiRu-1; Figure S4: PLS-DA score plots of the aliphatic region of the three cell lines treated with DiRu-1; Table S3: Summary of the parameters and statistical results of PLS-DA shown in Figure S4; Figure S5: PLS analysis score plots of the aromatic region of the three cell lines treated with DiRu-1; Figure S6: PCA loading plots for the PCA shown in Figure 3; Figure S7: PLS loading plots for the latent variables (LV1-3) of the PLS plots shown in Figure 3; Figure S8: PLS-DA loading plots for the latent variables of the PLS-DA plots shown in Figure S4; Figure S9: PCA loading plots of the PCA shown in S3, Figure S10: PLS loading plots for the latent variables of the PLS plots shown in Figure S5; Figure S11: TOCSY spectra of HEK-293 cells treated with a low dose (0.015 µM) of DiRu-1; Figure S12: TOCSY spectra of A2780 cells untreated; Figure S13: TOCSY spectra of A2780cisR cells treated with a high dose (0.03 µM) of DiRu-1; Figure S14: Percental change in the aliphatic buckets in A2780cisR cells: complete including unidentified buckets; Figure S15: Percental change in the aliphatic buckets in A2780 cells: complete including unidentified buckets; Figure S16: Percental change in the aliphatic buckets in HEK-293 cells: complete including unidentified buckets; Figure S17: Percental change in the aromatic buckets in A2780cisR cells; Figure S18: Percental change in the aromatic buckets in A2780 cells; Figure S19: Percental change in the aromatic buckets in HEK-293 cells; Figure S20: Comparison of bucket means for aliphatic region of A2780cisR; Figure S21: Comparison of bucket means for aliphatic region of A2780; Figure S22: Comparison of bucket means for aliphatic region of HEK-293; Figure S23: Comparison of bucket means for aromatic region of A2780cisR; Figure S24: Comparison of bucket means for aromatic region of A2780; and Figure S25: Comparison of bucket means for aromatic region of HEK-293.
