# Supplementary material for: 1H HR-MAS NMR-Based Metabolomics of Cancer Cells in Response to Treatment with the Diruthenium Trithiolato Complex [(p-MeC6H4iPr)2Ru2(SC6H4-p-But)3]+ (DiRu-1)
Source: Metabolites. 2019 Jul 18;9(7):146. doi: 10.3390/metabo9070146 (PMC6680816; doi:10.3390/metabo9070146)
Supplement: Supplementary file 1 [file metabolites-09-00146-s001.pdf]

Supporting Information: List of content

Supporting Figures

Figure S1. One-dimensional ^{1}H NMR PROJECT spectra of untreated (**a**) HEK-293, (**b**) A2780, and (**c**) A2780cisR cells.

Figure S2. Aromatic region of PROJECT spectra of the three untreated cells lines.

Figure S3. PCA score plots of the aromatic region of (**a**) HEK-293, (**b**,**c**) A2780, and (**d**) A2780cisR cells treated with 0.03 µM of DiRu-1 (blue), 0.015 of µM DiRu-1 (green), or the control (dark red).

Figure S4. PLS-DA score plots of the aliphatic region of (**a**) HEK-293, (**b**) A2780, and (**c**) A2780cisR cells treated with 0.03 µM of DiRu-1 (blue) and the untreated control (dark red).

Figure S5. PLS analysis score plots of the aromatic region of (**a**) HEK-293, (**b**) A2780, and (**c**) A2780cisR cells treated with 0.03 µM of DiRu-1 (blue), 0.015 µM of DiRu-1 (green), or the untreated control (dark red).

Figure S6. PCA loading plots for the PCA shown in Figure 3 (aliphatic region) of (**a**–**c**) HEK-293, (**d**–**f**) A2780, and (**g**,**h**) A2780cisR cells. Control, low dose (0.015 µM), and full dose (0.03 µM) were analysed.

Figure S7. PLS loading plots for the latent variables (LV1–3) of the PLS plots shown in Figure 3 (aliphatic region) of (**a**,**b**) HEK-293, (**c**–**e**) A2780, and (**f**,**g**) A2780cisR cells: Control, low dose (0.015 µM), and full dose (0.03 µM) were analysed.

Figure S8. PLS-DA loading plots for the latent variables of the PLS-DA plots shown in Figure S6 (aliphatic region) of (**a**,**b**) HEK-293, (**c**–**e**) A2780, and (**f**–**h**) A2780cisR cells: Only control and full dose (0.03 µM) were analysed.

Figure S9. PCA loading plots of the PCA shown in S3 (aromatic region) of (**a**–**c**) HEK-293, (**d**,**e**) A2780, and (**f**–**h**) A2780cisR cells: Control, low dose (0.015 µM), and full dose (0.03 µM) were analysed.

Figure S10. PLS loading plots for the latent variables of the PLS plots shown in Figure S8 (aromatic region) of (**a**–**c**) HEK-293, (**d**,**e**) A2780, and (**f**–**h**) A2780cisR cells: Control, low dose (0.015 µM), and full dose (0.03 µM) were analysed.

Figure S11. TOCSY spectra of HEK-293 cells treated with a low dose (0.015 µM) of DiRu-1.

Figure S12. TOCSY spectra of A2780 cells untreated.

Figure S13. TOCSY spectra of A2780cisR cells treated with a high dose (0.03 µM) of DiRu-1.

Figure S14. Percental change in the aliphatic buckets in A2780cisR cells: complete including unidentified buckets.

Figure S15. Percental change in the aliphatic buckets in A2780 cells: complete including unidentified buckets.

Figure S16. Percental change in the aliphatic buckets in HEK-293 cells: complete including unidentified buckets.

Figure S17. Percental change in the aromatic buckets in A2780cisR cells.

Figure S18. Percental change in the aromatic buckets in A2780 cells.

Figure S19. Percental change in the aromatic buckets in HEK-293 cells.

Figure S20. Comparison of bucket means for aliphatic region of A2780cisR.

Figure S21. Comparison of bucket means for aliphatic region of A2780.

Figure S22. Comparison of bucket means for aliphatic region of HEK-293.

Figure S23. Comparison of bucket means for aromatic region of A2780cisR.

Figure S24. Comparison of bucket means for aromatic region of A2780.

Figure S25. Comparison of bucket means for aromatic region of HEK-293.

Supporting Tables

Table S1. The 52 buckets in the aliphatic region of the 1-D ^{1}H NMR spectra (0.79–4.40 ppm) and the corresponding metabolites

Table S2. The 44 buckets in the aromatic region of the 1-D 1H NMR spectra (5.78–8.95 ppm) and the corresponding metabolites. (The metabolites in brackets can be present partially.)

Table S3. Summary of parameters and statistical results of PLS-DA shown in Figure S6.

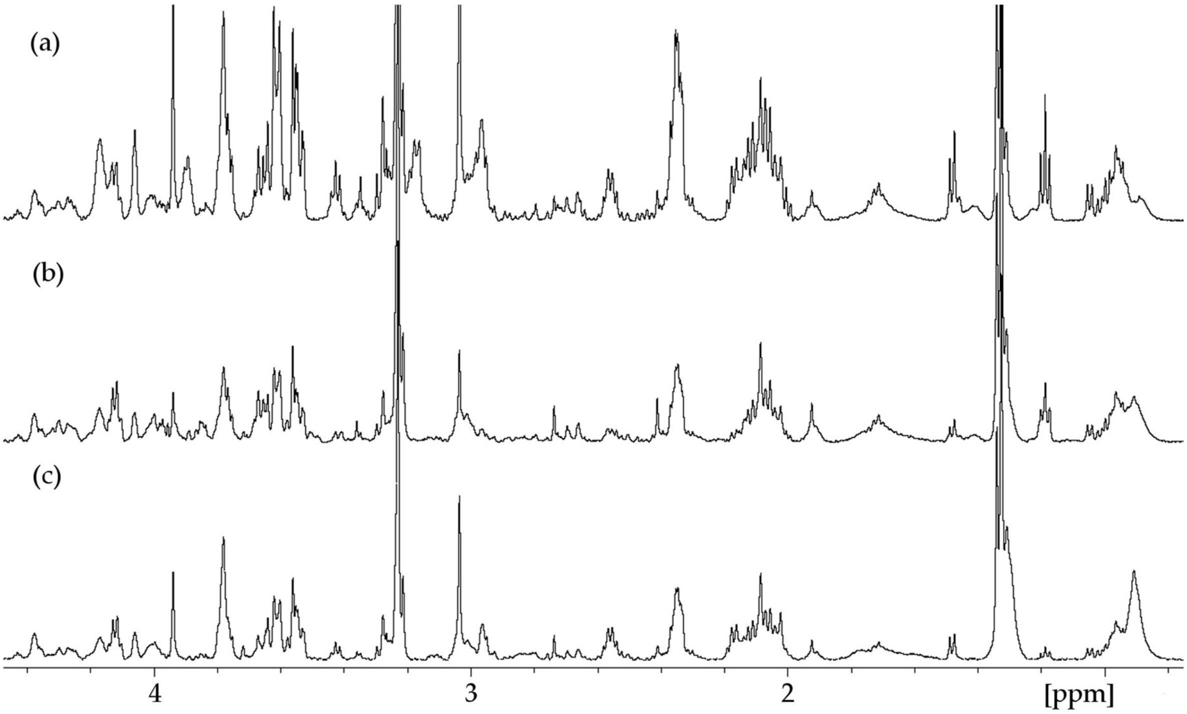

Figure S1. One-dimensional ^{1}H NMR PROJECT spectra of untreated (**a**) HEK-293, (**b**) A2780, and (**c**) A2780cisR cells.

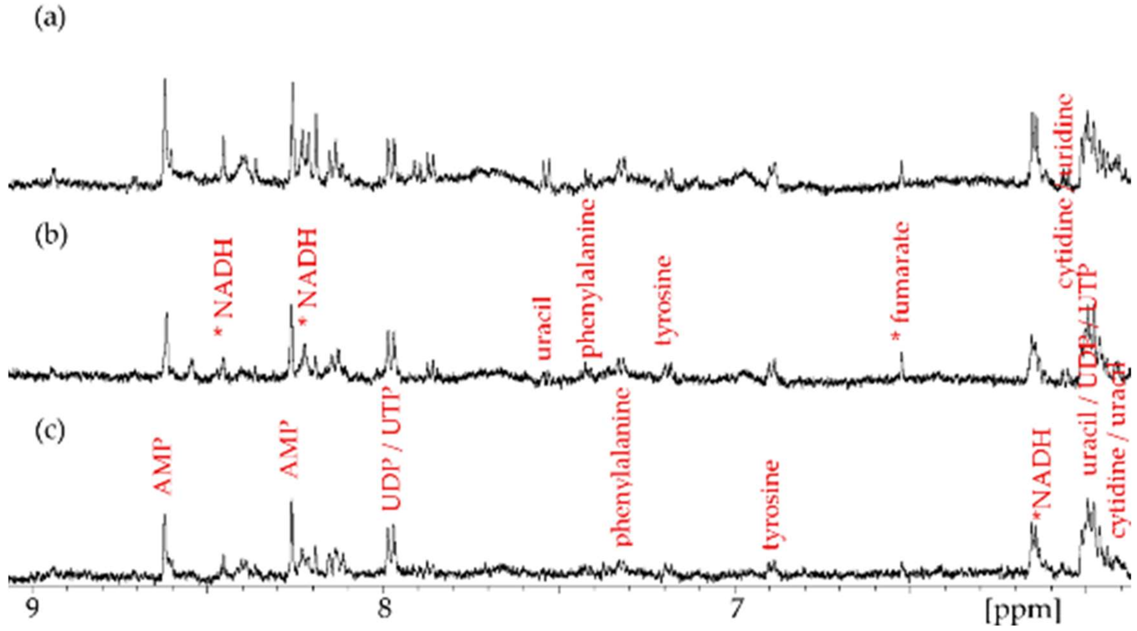

Figure S2. Aromatic region of PROJECT spectra of the three untreated cells lines: (**a**) HEK-293, (**b**) A2780, and (**c**) A2780cisR.

Table S1. The 52 buckets in the aliphatic region of the 1-D ^{1}H NMR spectra (0.79–4.40 ppm) and the corresponding metabolites. (The metabolites in brackets can be present partially.) Ac = acetate; Ala = alanine; Chol = choline; Cre = creatine; Cys = cysteine; Glu = glutamate; Gln = glutamine; GSH = glutathione; Lac = lactate; Leu = leucine; myo-Ino = myo-inositol; = phosphocholine; Tyr = tyrosine.

Bucket	Range (ppm)	Metabolites
1	0.791–0.991	lipid ω-CH_3, Leu
2	0.991–1.029	not assigned
3	1.029–1.069	Val
4	1.227–1.318	lipid (–CH_2)n
5	1.318–1.361	lipid (–CH_2)n, Lac
6	1.361–1.465	not assigned
7	1.465–1.502	Ala
8	1.545–1.635	lipid β-CH_2
9	1.635–1.756	Leu
10	1.756–1.815	not assigned
11	1.880–1.946	Ac (+ other)
12	1.982–2.143	lipid –CH_2–CH=, Gln/Glu
13	2.153–2.198	GSH, Glu, Gln
14	2.219–2.287	lipid α-CH_2 (+ other)
15	2.291–2.321	not assigned
16	2.321–2.381	Glu
17	2.381–2.404	Glu
18	2.404–2.422	Glu , Gln
19	2.422–2.484	Gln
20	2.484–2.618	GSH
21	2.618–2.674	not assigned
22	2.674–2.731	not assigned
23	2.731–2.748	not assigned
24	2.748–2.764	not assigned
25	2.909–2.938	(GSH)
26	2.938–2.976	GSH
27	2.976–3.020	not assigned
28	3.022–3.051	Cre (+ possibly Cys)
29	3.075–3.203	Tyr, Cys
30	3.203–3.218	Chol
31	3.218–3.246	PC
32	3.246–3.304	myo-ino, Tyr
33	3.341–3.354	not assigned
34	3.354–3.368	not assigned
35	3.383–3.457	not assigned
36	3.471–3.519	not assigned
37	3.519–3.537	Chol, myo-ino
38	3.537–3.557	myo-ino (+ possibly Chol)
39	3.557–3.572	not assigned
40	3.572–3.587	not assigned
41	3.587–3.632	PC, myo-ino
42	3.732–3.816	GSH, Glu, Ala, (Gln)

43	3.816–3.871	not assigned
44	3.871–3.925	not assigned
45	3.925–3.951	Cre (+ Tyr)
46	3.951–3.987	Cys (+ Tyr)
47	3.987–4.044	Cys
48	4.044–4.077	Chol, myo-ino
49	4.093–4.148	Lac
50	4.148–4.200	PC
51	4.218–4.365	not assigned
52	4.365–4.400	not assigned

Table S2. The 44 buckets in the aromatic region of the 1-D 1H NMR spectra (5.78–8.95 ppm) and the corresponding metabolites. (The metabolites in brackets can be present partially.) AMP = adenosine monophosphate; Cyd = cytidine; Fum = fumarate; NADH = Nicotinamide adenine dinucleotide; Phe = phenylalanine; Tyr = tyrosine, UDP = uridine diphosphate; Ura = uracil; Urd = uridine; UTP = uridine triphosphate.

Bucket	Range (ppm)	Metabolites
1	5.786–5.818	Ura
2	5.86–5.898	not assigned
3	5.898–5.927	Cyt/Urd
4	5.927–5.946	not assigned
5	5.946–5.968	Ura/UDP/UTP
6	5.968–5.983	Ura/UDP/UTP
7	5.983–6.008	Ura/UDP/UTP
8	6.008–6.024	Ura/UDP/UTP
9	6.042–6.057	Cyt/Urd
10	6.057–6.076	not assigned
11	6.082–6.123	not assigned
12	6.123–6.17	NADH*
13	6.513–6.532	Fum*
14	6.875–6.893	Tyr
15	6.893–6.911	Tyr
16	7.085–7.125	not assigned
17	7.125–7.17	not assigned
18	7.17–7.208	Tyr
19	7.284–7.302	not assigned
20	7.302–7.346	Phe
21	7.346–7.399	not assigned
22	7.399–7.451	Phe
23	7.52–7.536	Ura
24	7.536–7.554	Ura
25	7.554–7.612	not assigned
26	7.846–7.864	not assigned
27	7.864–7.882	not assigned
28	7.886–7.903	not assigned
29	7.903–7.922	not assigned
30	7.922–7.957	not assigned

31	7.957–7.996	UDP/UTP
32	7.996–8.027	UDP
33	8.081–8.1	not assigned
34	8.1–8.179	not assigned
35	8.182–8.201	NADH*
36	8.201–8.245	NADH*
37	8.245–8.275	AMP
38	8.354–8.371	not assigned
39	8.371–8.426	not assigned
40	8.446–8.462	NADH*
41	8.529–8.558	not assigned
42	8.594–8.637	AMP
43	8.691–8.725	not assigned
44	8.923–8.952	not assigned

*tentative assignment

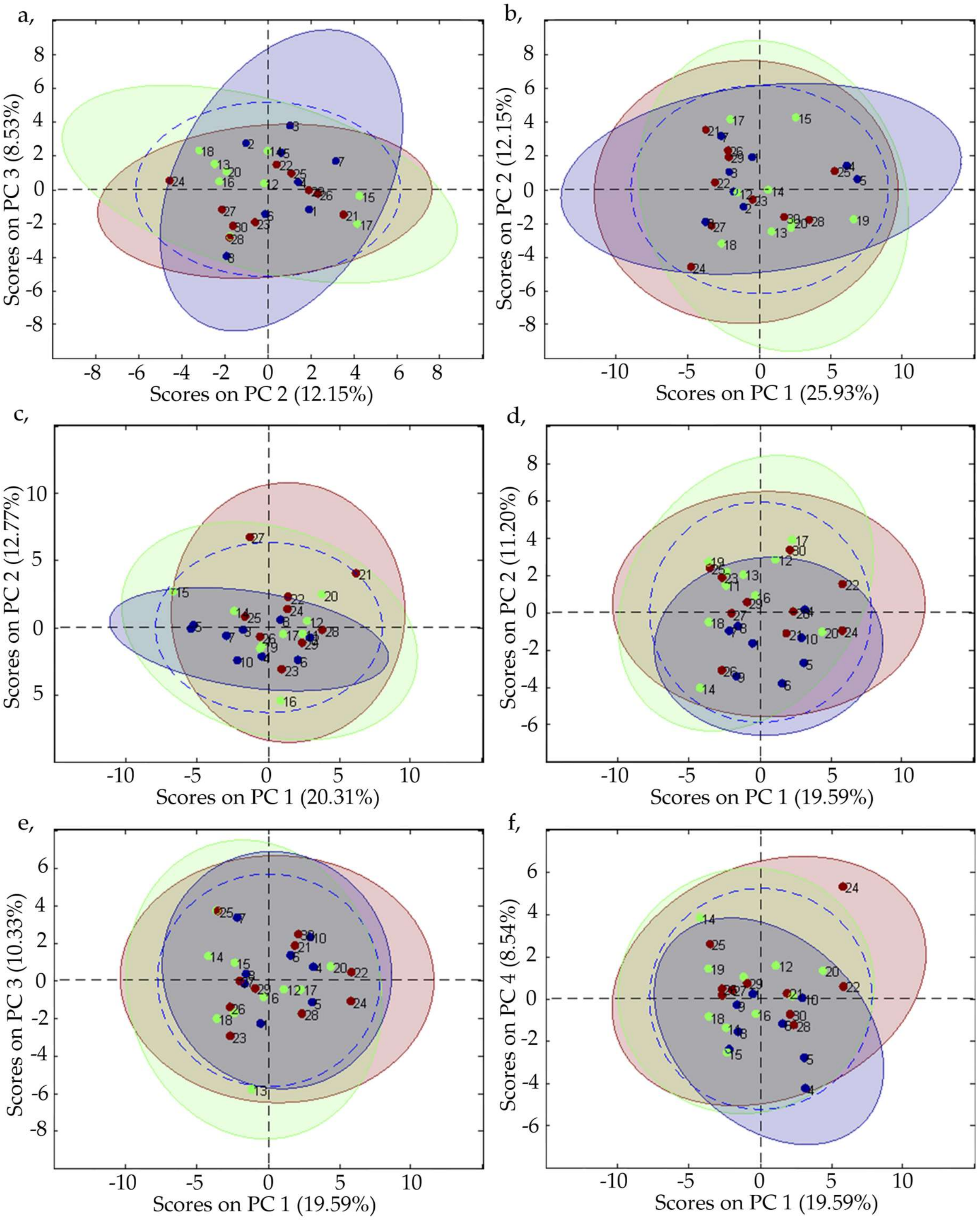

Figure S3. PCA score plots of the aromatic region of (**a**) HEK-293, (**b**,**c**) A2780, and (**d**) A2780cisR cells treated with 0.03 μM of DiRu-1 (blue), 0.015 of μM DiRu-1 (green), or the control (dark red).

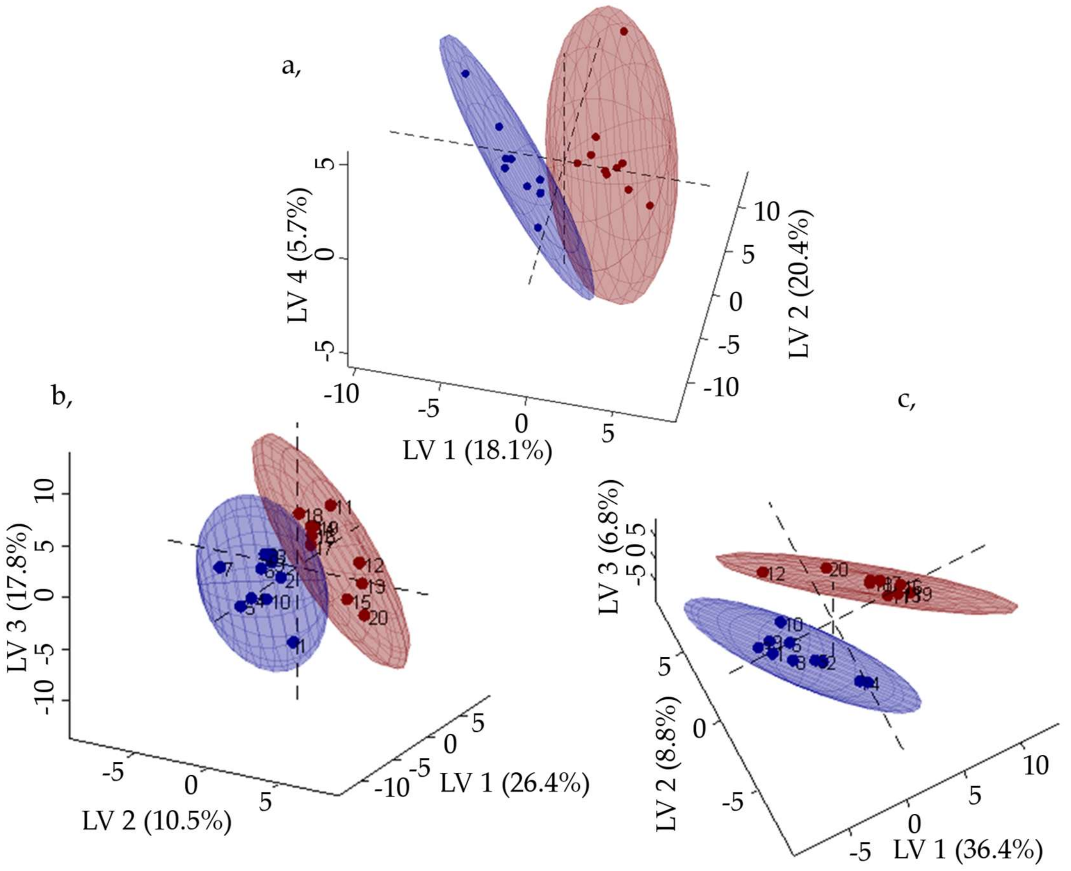

Figure S4. PLS-DA score plots of the aliphatic region of (**a**) HEK-293, (**b**) A2780, and (**c**) A2780cisR cells treated with 0.03 µM of DiRu-1 (blue) and the untreated control (dark red).

Table S3: Summary of parameters and statistical results of PLS-DA shown in Figure S6.

**Groups**	**HEK-293**	**A2780**	**A2780cisR**
**X-block**	20 x 52	20 x 52	19 x 52
**Number of LVs**	4	5	3
**Included samples**	(1–20) (1–52)	(1–20) (1–52)	(1–13 15–20) (1–52)
**Preprocessing**	mean center, UVS	mean center, UVS	mean center, UVS
**Algorithm**	PLS-DA	PLS-DA	PLS-DA
**Cross validation**	Venetian blinds 4 splits, 1 sample/split	Venetian blinds 4 splits, 1 sample/split	Venetian blinds 4 splits, 1 sample/split
**RMSEC**	0.088	0.079	0.100
**RMSECV**	0.318	0.542	0.231
**R^2Cal**	0.969	0.975	0.960
**R^2CV**	0.601	0.055	0.788
**Wilcoxon (self-pred.)* y-column 1**	0.025	0.026	0.010
**Wilcoxon (self-pred.)* y-column 2**	0.025	0.026	0.010
**Wilcoxon (cross-val.)* y-column 1**	0.021	0.162	0.002
**Wilcoxon (cross-val.)* y-column 2**	0.021	0.162	0.002
**Sign test (self-pred.)* y-column 1**	0.081	0.089	0.036

**Sign test (self-pred.)* y-column 2**	0.081	0.089	0.036
**Sign test (cross-val.)* y-column 1**	0.082	0.263	0.011
**Sign test (cross-val.)* y-column 2**	0.082	0.263	0.011
**Rand t-test (self-pred.)* y-column 1**	0.043	0.056	0.060
**Rand t-test (self-pred.)* y-column 2**	0.043	0.052	0.061
**Rand t-test (cross-val.)* column 1**	0.021	0.207	0.007
**Rand t-test (cross-val.)* column 2**	0.020	0.212	0.008
**Total variance captured**	50.90%	66.50%	52.04%
**LV 1**	18.12%	26.43%	36.43%
**LV 2**	20.37%	10.51%	8.78%
**LV 3**	6.73%	17.83%	6.83%
**LV 4**	5.68%	5.86%	
**LV 5**		5.88%	
* Values bellow 0.05 indicate significance at 95% confidence level.			

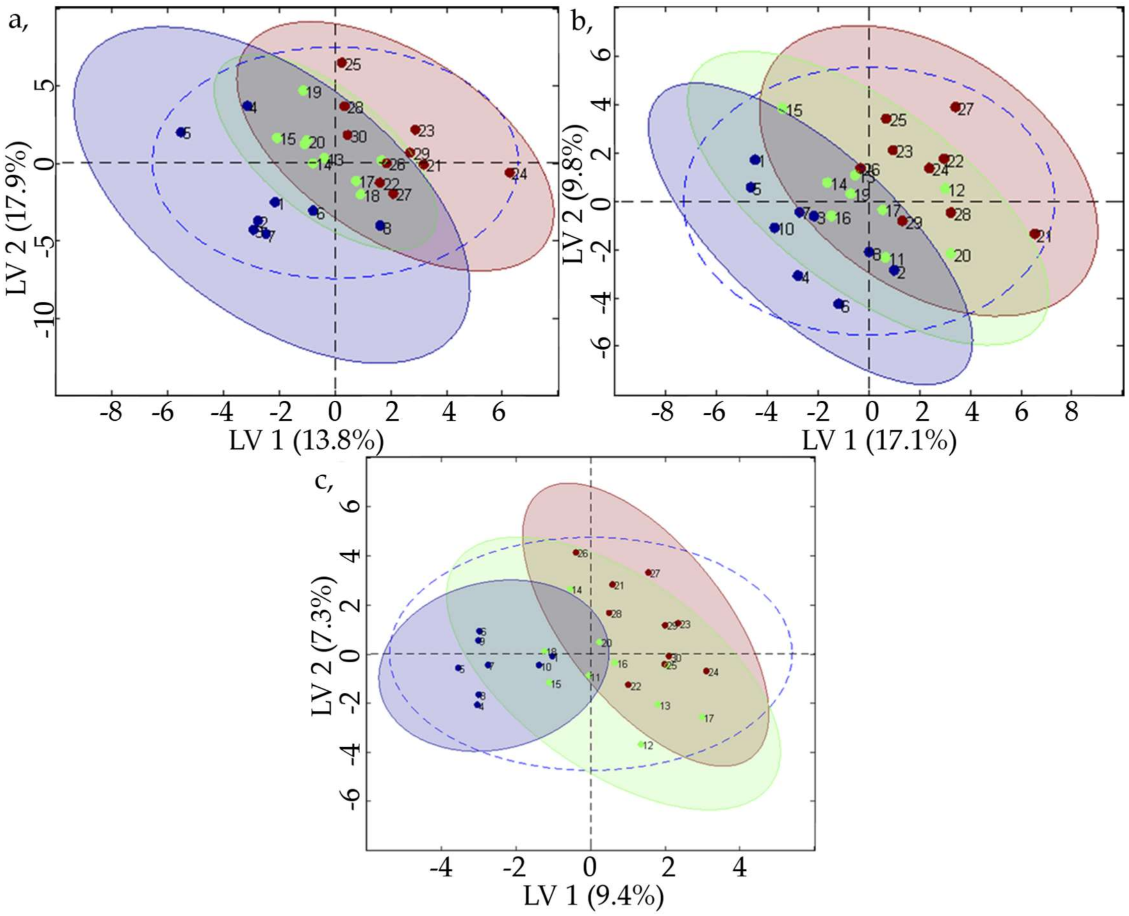

Figure S5. PLS analysis score plots of the aromatic region of (**a**) HEK-293, (**b**) A2780, and (**c**) A2780cisR cells treated with 0.03 μM of DiRu-1 (blue), 0.015 μM of DiRu-1 (green), or the untreated control (dark red).

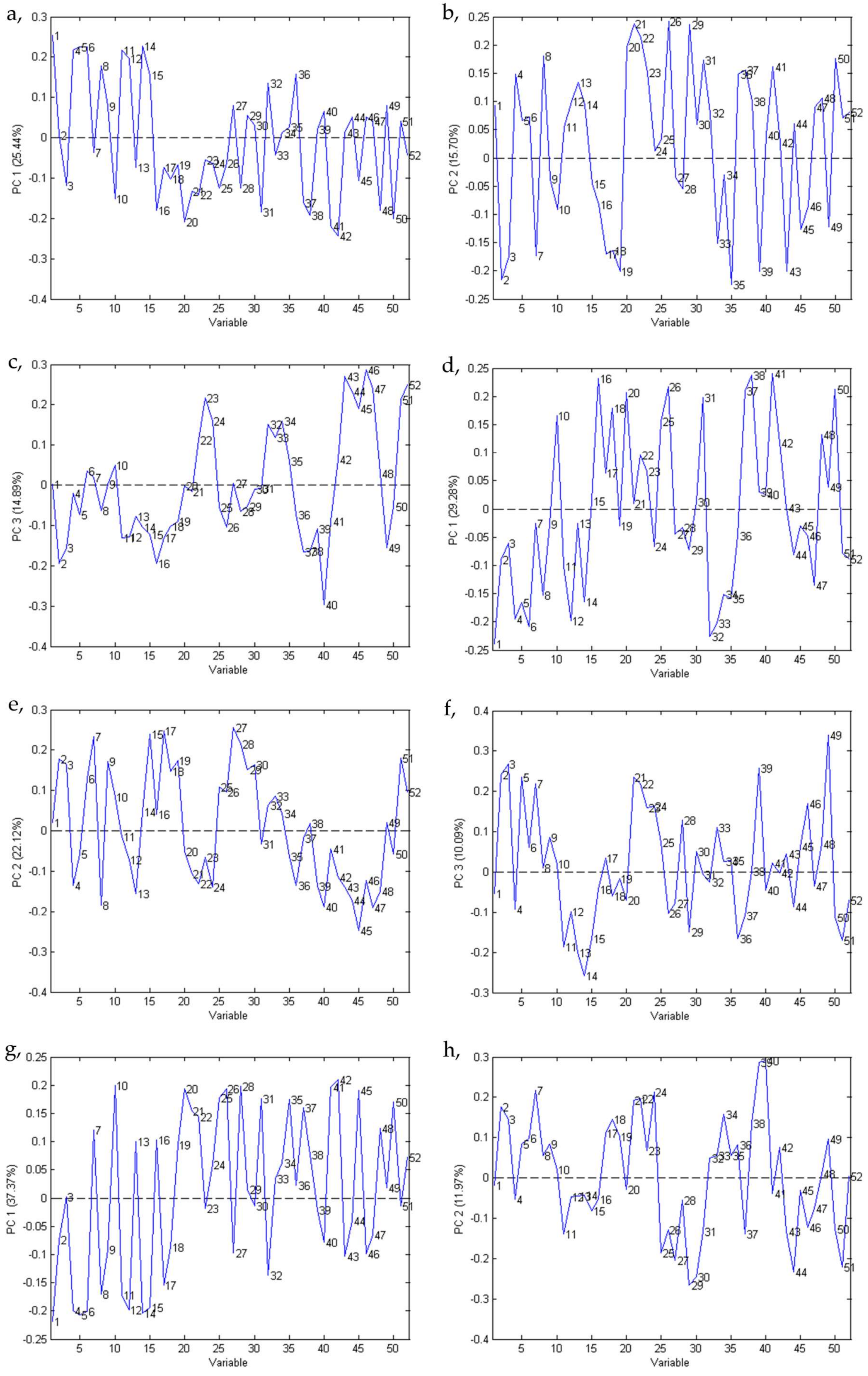

Figure S6. PCA loading plots for the PCA shown in Figure 3 (aliphatic region) of (**a**–**c**) HEK-293, (**d**–**f**) A2780, and (**g**,**h**) A2780cisR cells. Control, low dose (0.015 µM), and full dose (0.03 µM) were analysed.

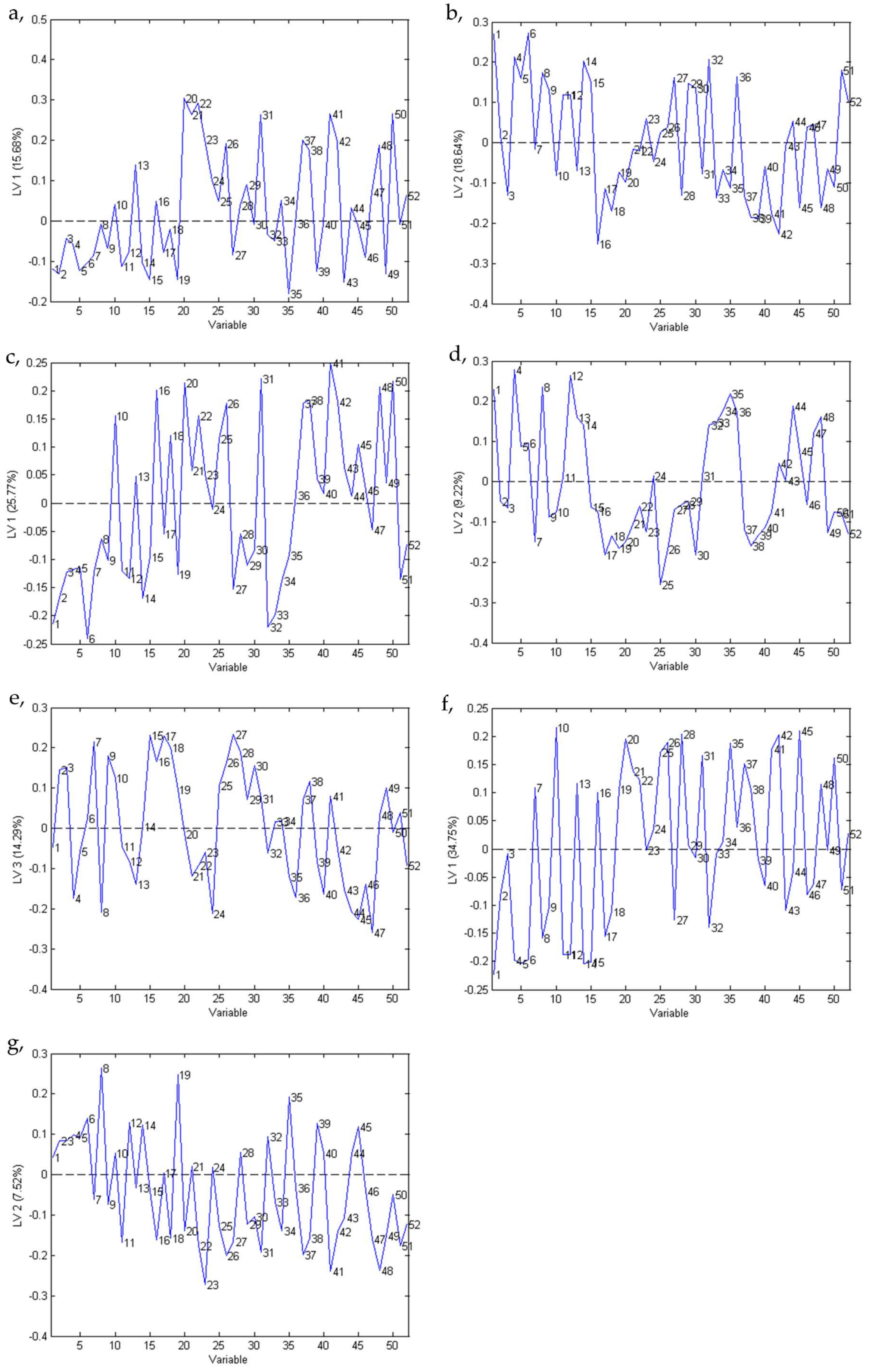

Figure S7. PLS loading plots for the latent variables (LV1–3) of the PLS plots shown in Figure 3 (aliphatic region) of (**a**,**b**) HEK-293, (**c**–**e**) A2780, and (**f**,**g**) A2780cisR cells: Control, low dose (0.015 μM), and full dose (0.03 μM) were analysed.

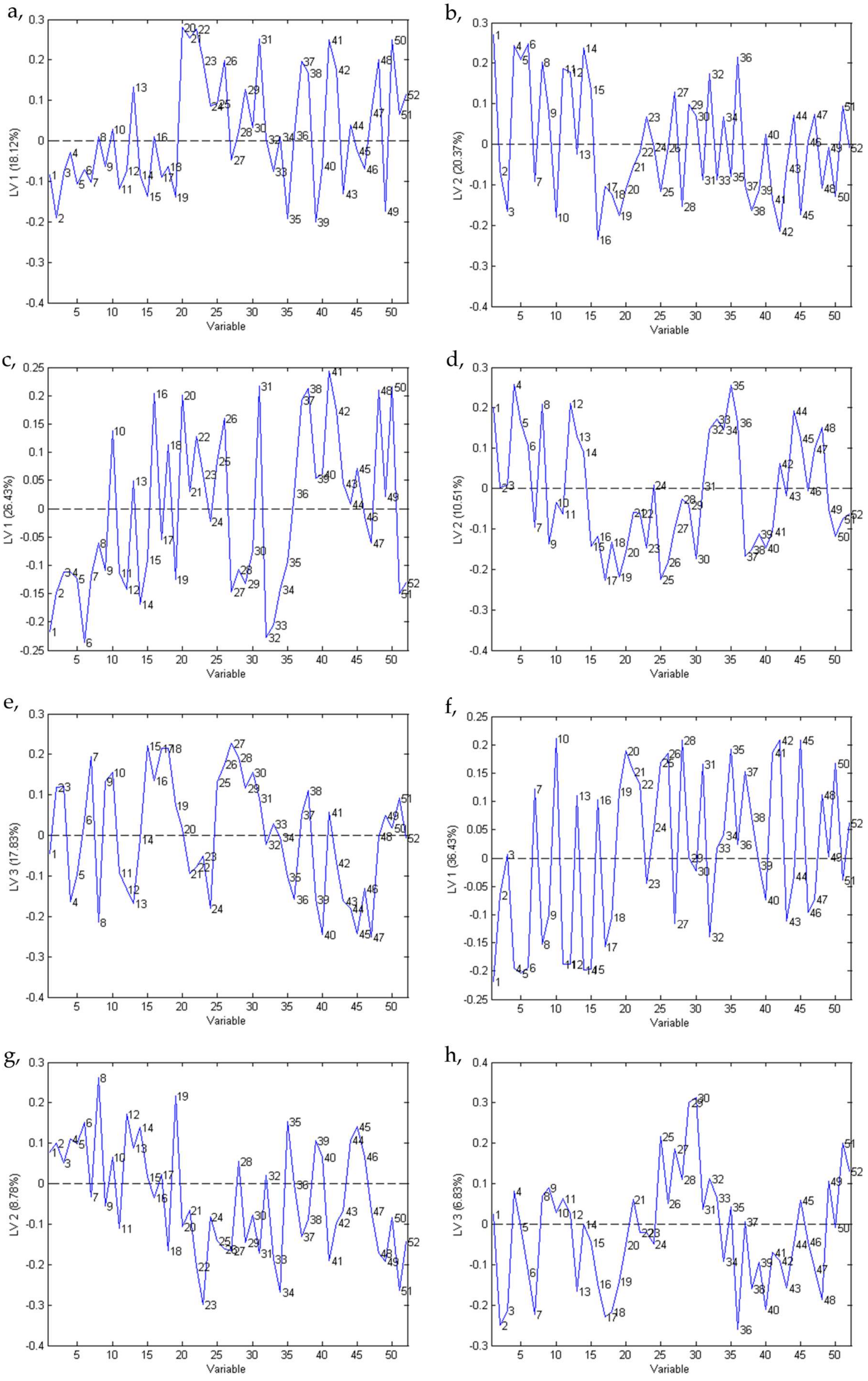

Figure S8. PLS-DA loading plots for the latent variables of the PLS-DA plots shown in Figure S6 (aliphatic region) of (**a**,**b**) HEK-293, (**c**–**e**) A2780, and (**f**–**h**) A2780cisR cells: Only control and full dose (0.03 μM) were analysed.

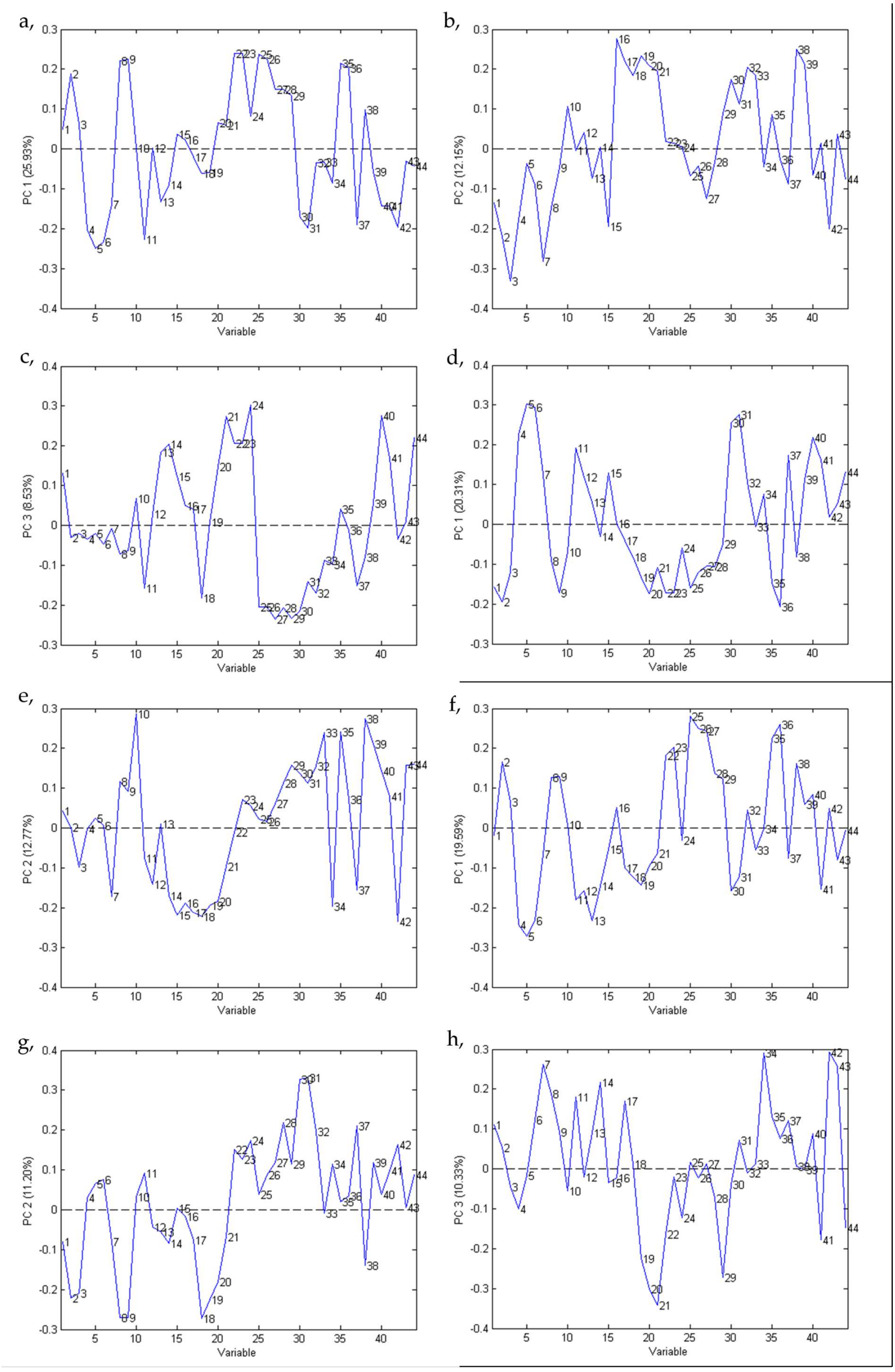

Figure S9. PCA loading plots of the PCA shown in S5 (aromatic region) of (**a**–**c**) HEK-293, (**d**,**e**) A2780, and (**f**–**h**) A2780cisR cells: Control, low dose (0.015 μM), and full dose (0.03 μM) were analysed.

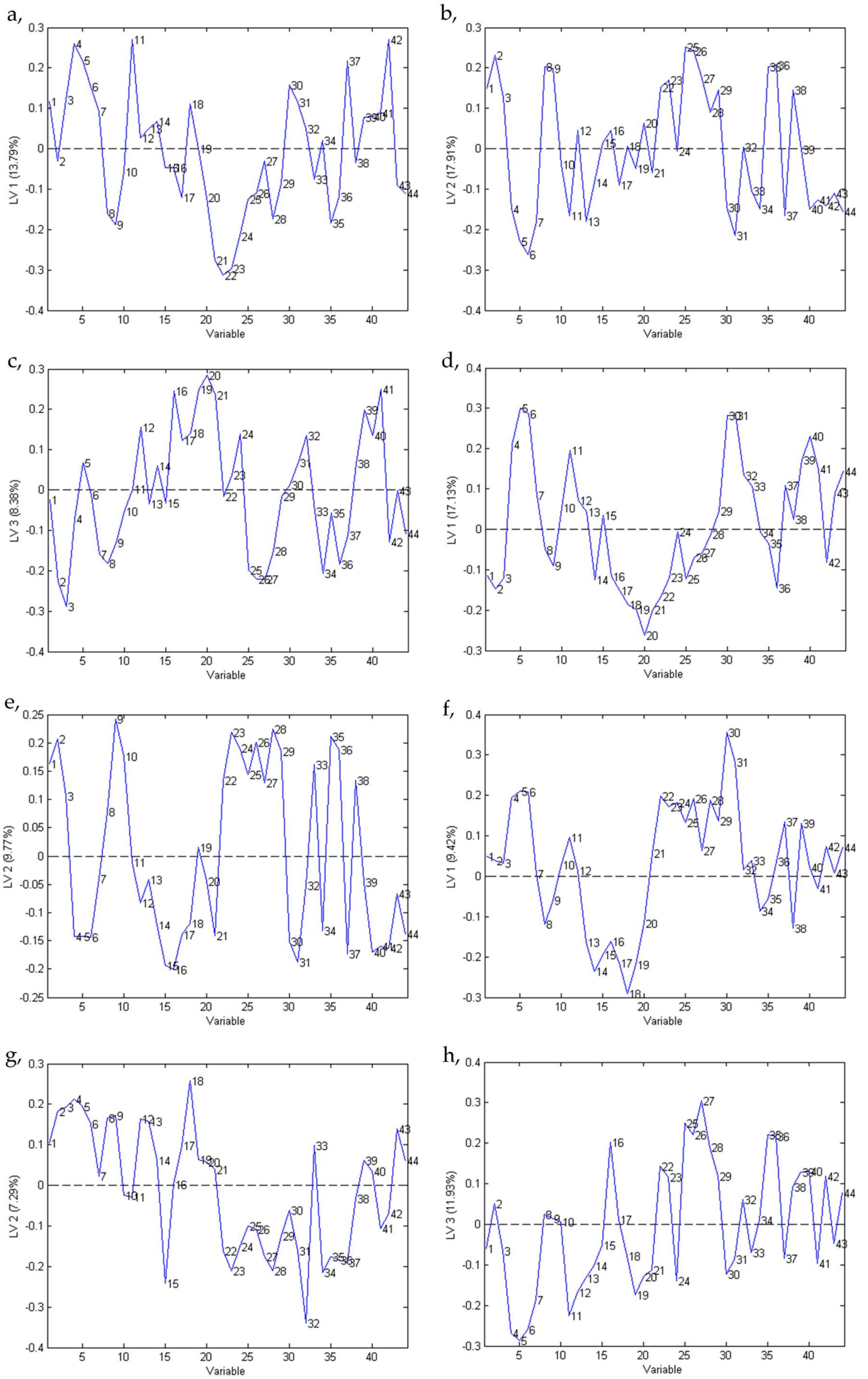

Figure S10. PLS loading plots for the latent variables of the PLS plots shown in Figure S8 (aromatic region) of (**a**–**c**) HEK-293, (**d**,**e**) A2780, and (**f**–**h**) A2780cisR cells: Control, low dose (0.015 μM), and full dose (0.03 μM) were analysed.

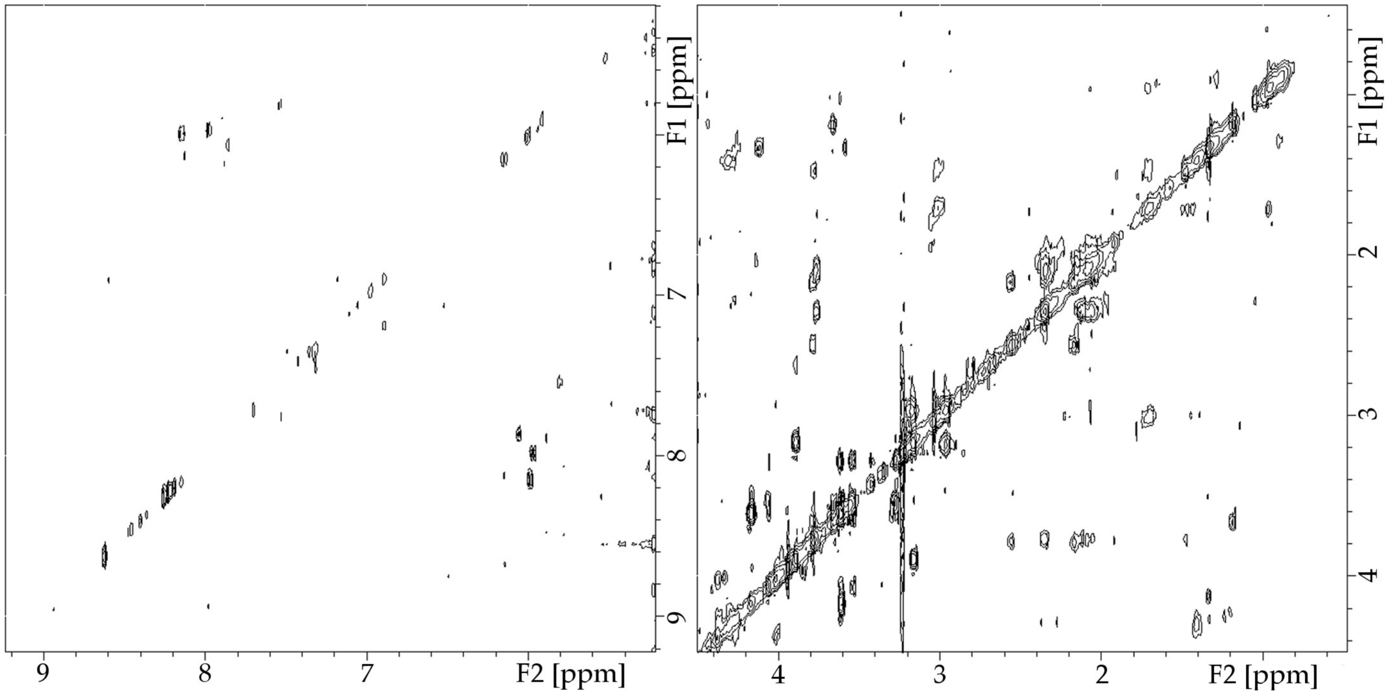

Figure S11. TOCSY spectra of HEK-293 cells treated with a low dose (0.015 μM) of DiRu-1.

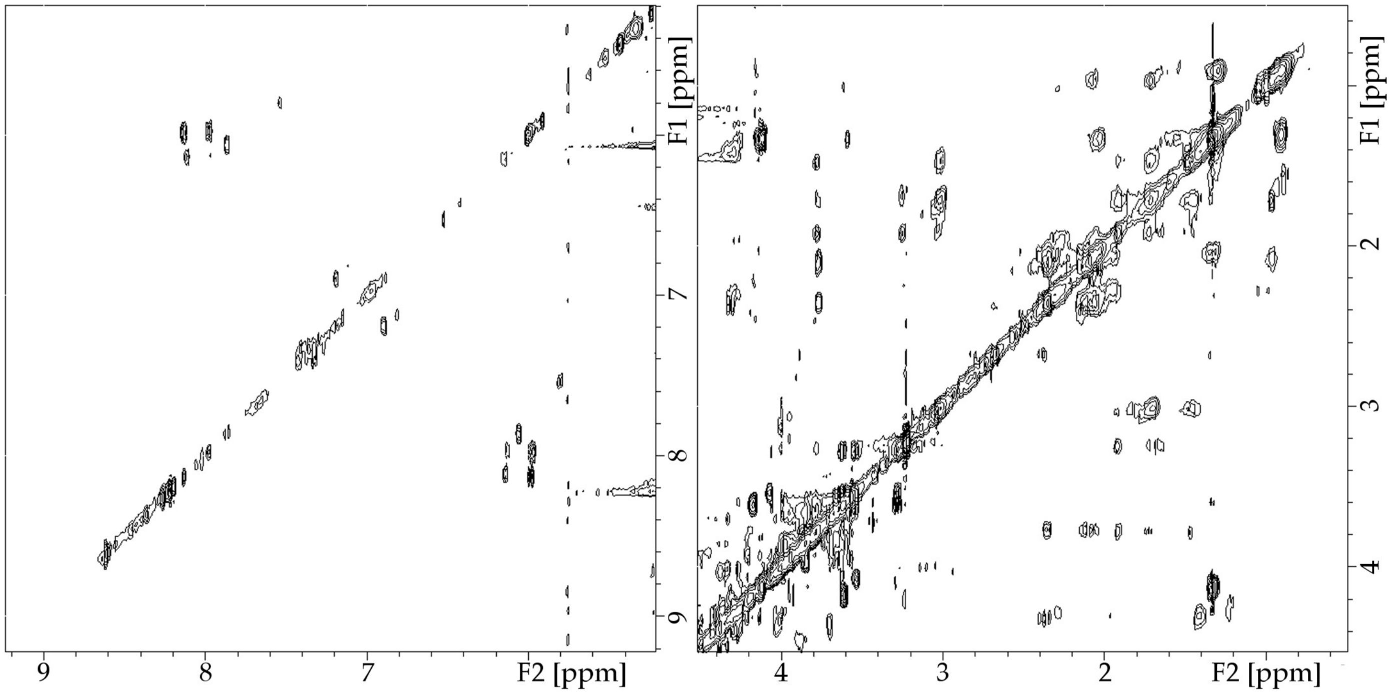

Figure S12. TOCSY spectra of A2780 cells untreated.

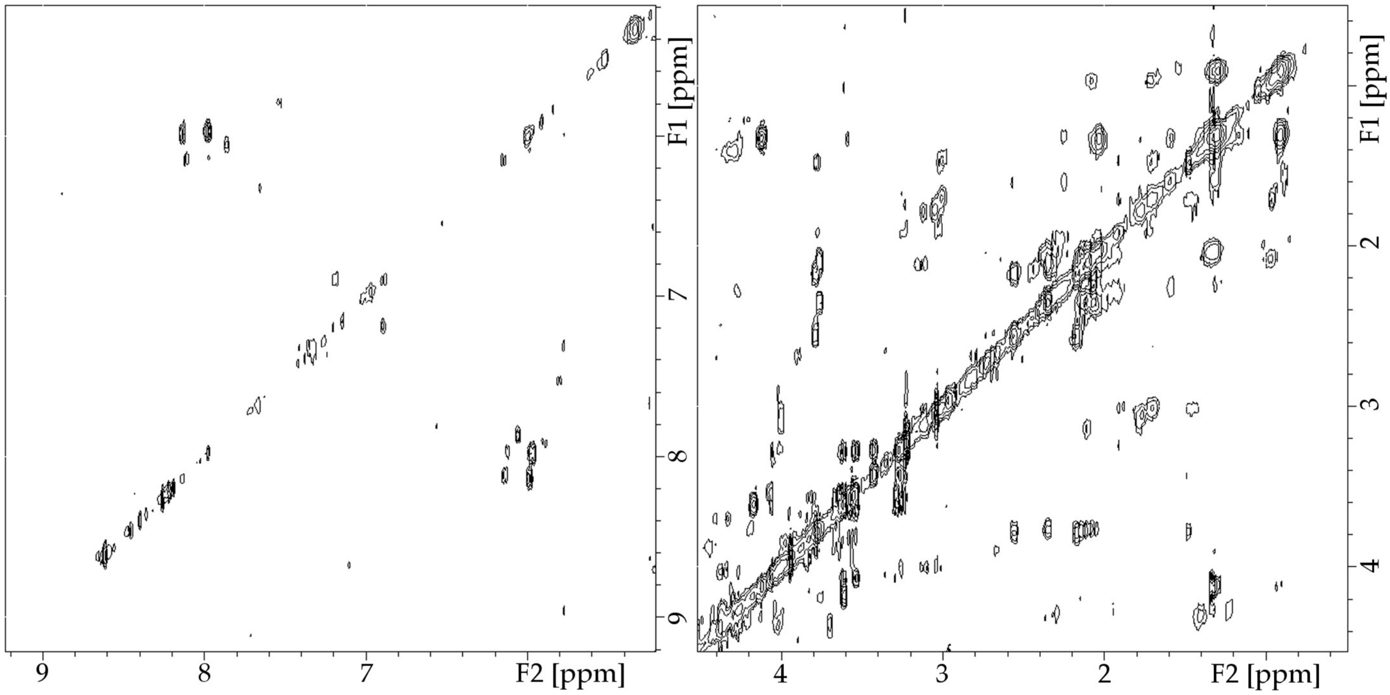

Figure S13. TOCSY spectra of A2780cisR cells treated with a high dose (0.03 μM) of DiRu-1.

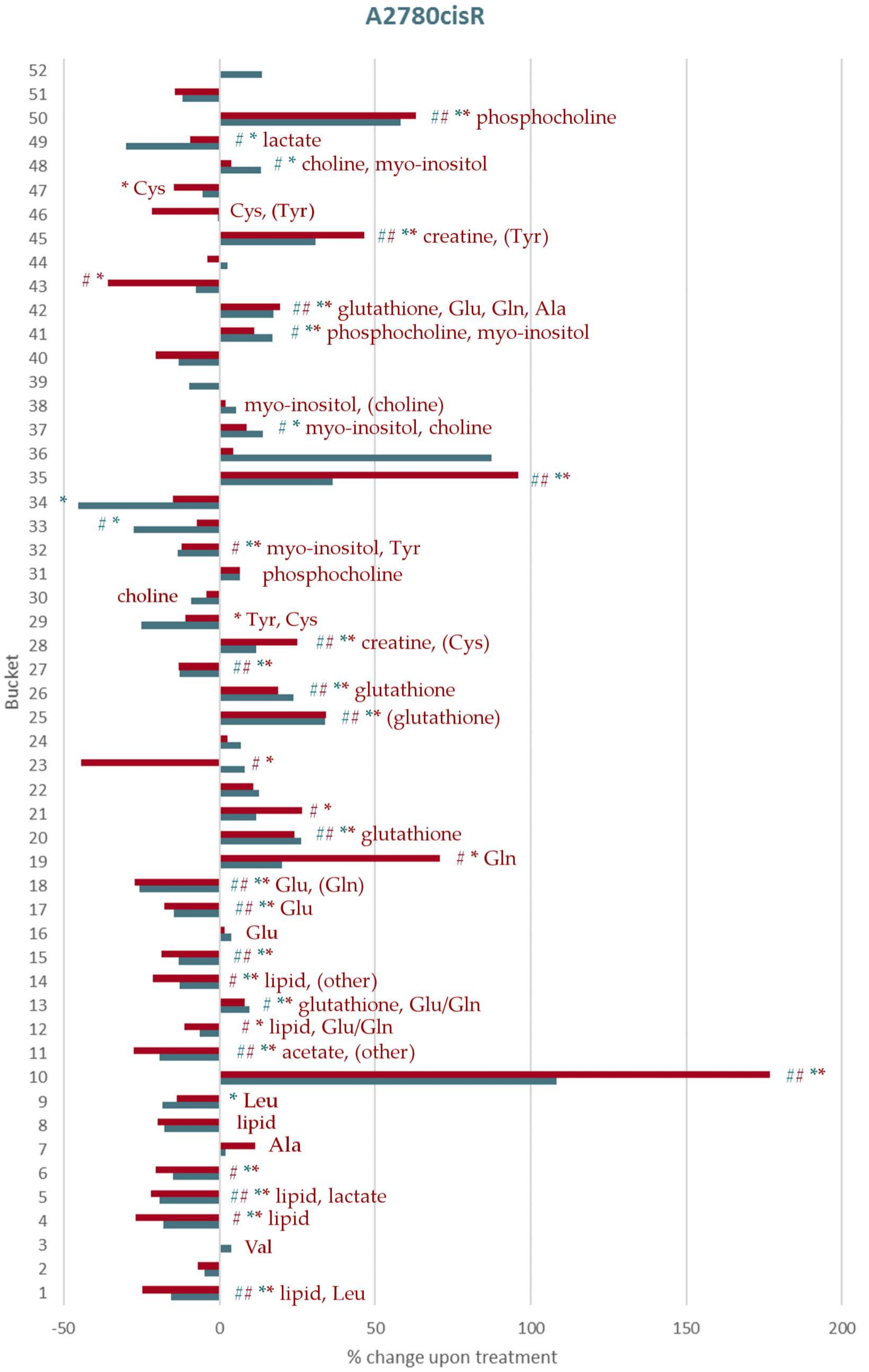

Figure S14. Percental change in the aliphatic buckets in A2780cisR cells: The increasing/decreasing level of each bucket compared to the control in cells treated with 0.015 µM DiRu-1 (blue) and 0.03 µM DiRu-1 (red) are expressed in percentage. The buckets marked with * show *p*-values < 0.05. The buckets marked with # show Benjamini–Hochberg corrected *p*-values < 0.05.

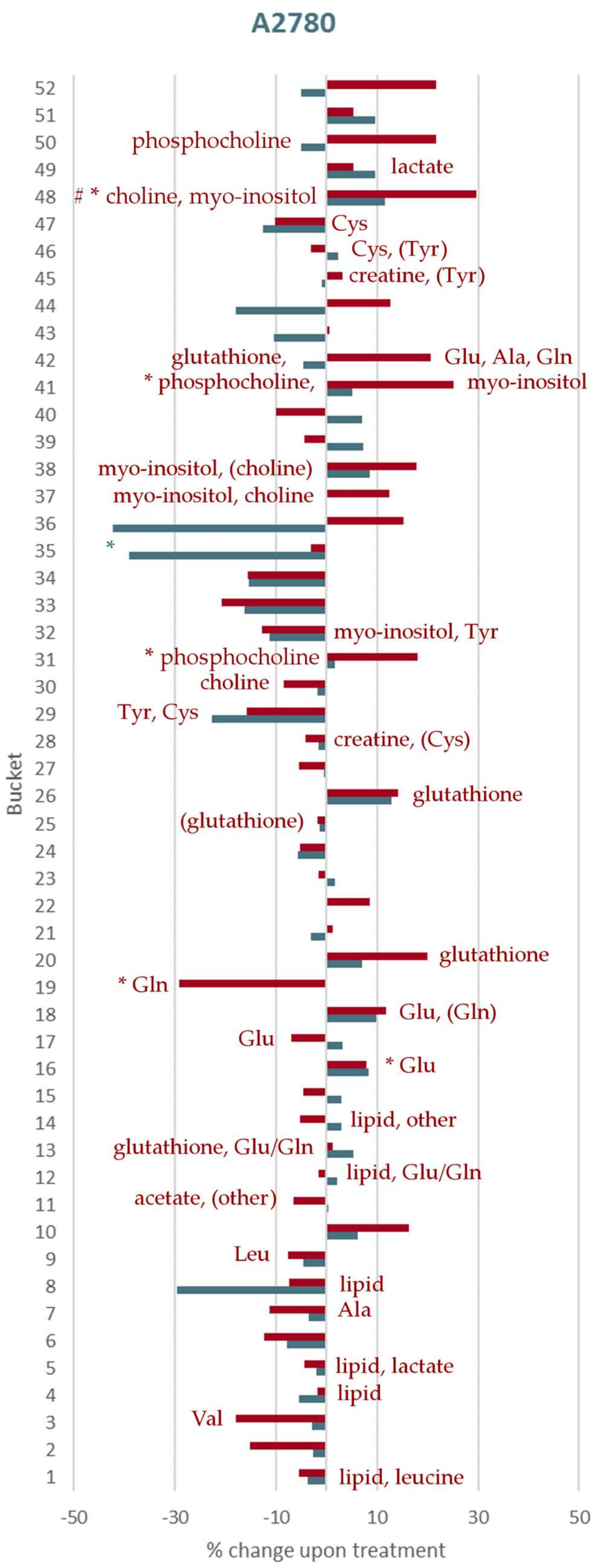

Figure S15. Percental change in the aliphatic buckets in A2780 cells: The increasing/decreasing level of each bucket compared to the control in cells treated with 0.015 µM DiRu-1 (blue) and 0.03 µM DiRu-1 (red) are expressed in percentage. The buckets marked with * show p-values < 0.05. The buckets marked with # show Benjamini–Hochberg corrected p-values < 0.05.

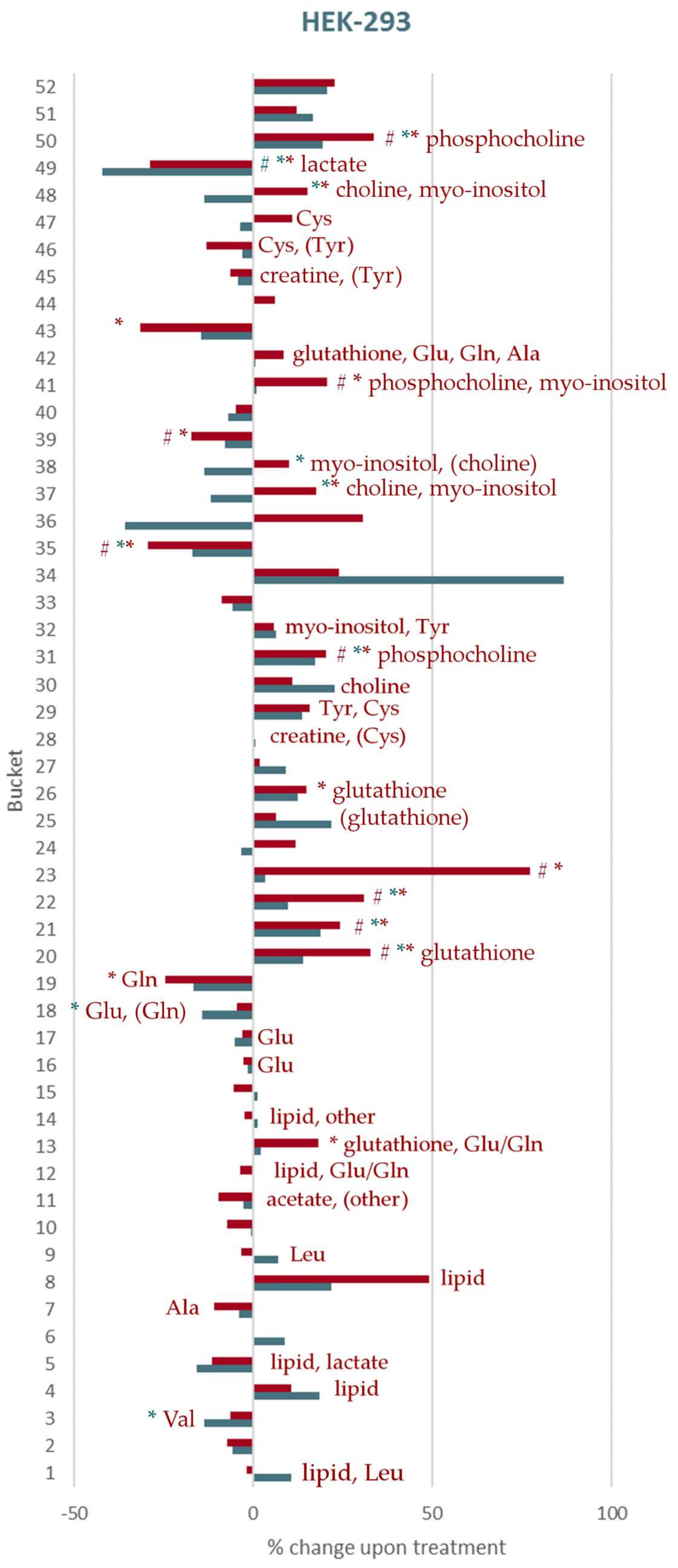

Figure S16. Percental change in the aliphatic buckets in HEK-293 cells: The increasing/decreasing level of each bucket compared to the control in cells treated with 0.015 µM DiRu-1 (blue) and 0.03 µM DiRu-1 (red) are expressed in percentage. The buckets marked with * show p-values < 0.05. The buckets marked with # show Benjamini–Hochberg corrected p-values < 0.05.

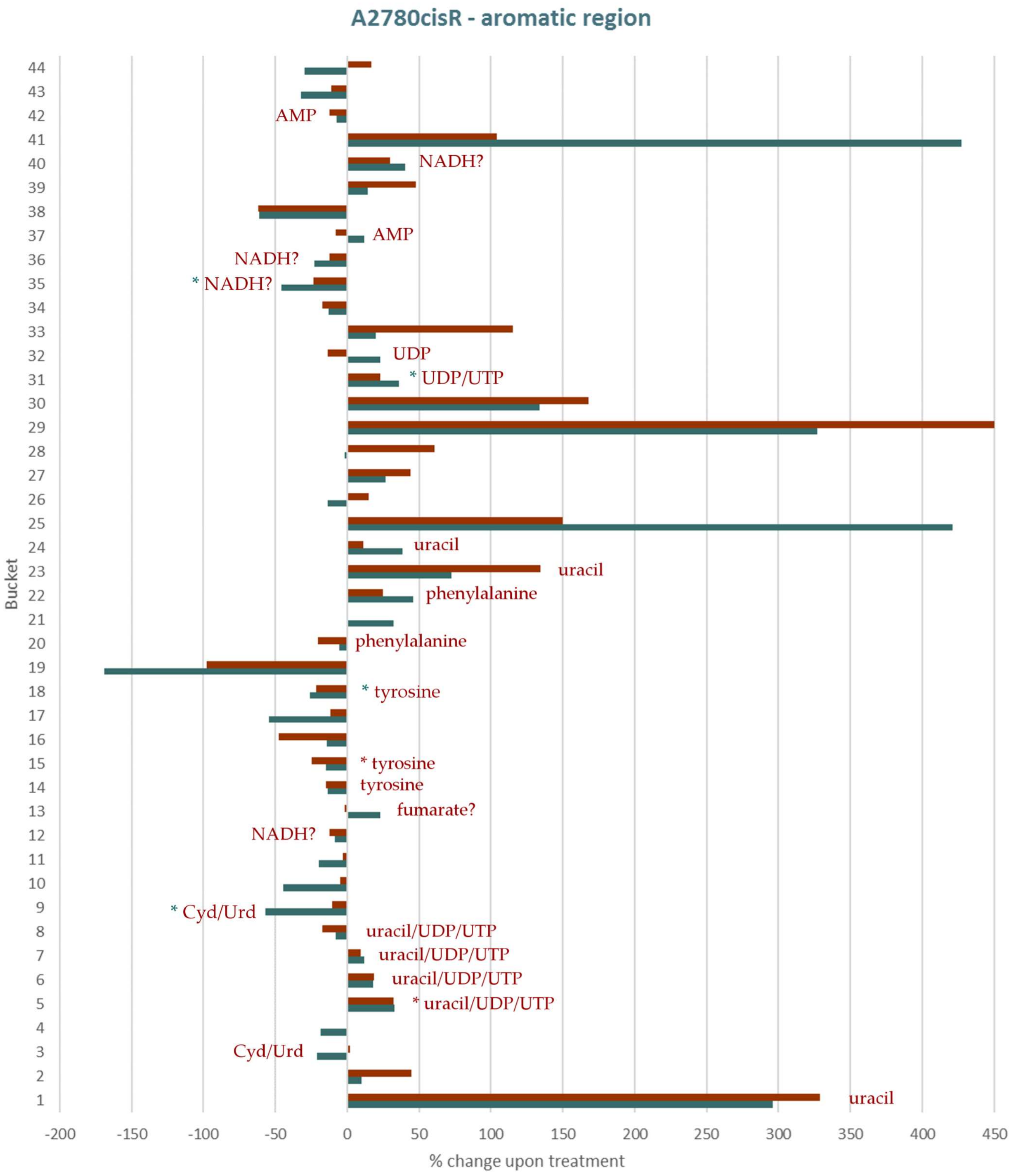

Figure S17. Percental change in the aromatic buckets in A2780cisR cells: The increasing/decreasing level of each bucket compared to the control in cells treated with 0.015 µM DiRu-1 (turquoise) and 0.03 µM DiRu-1 (red) are expressed in percentage. * p-value < 0.05

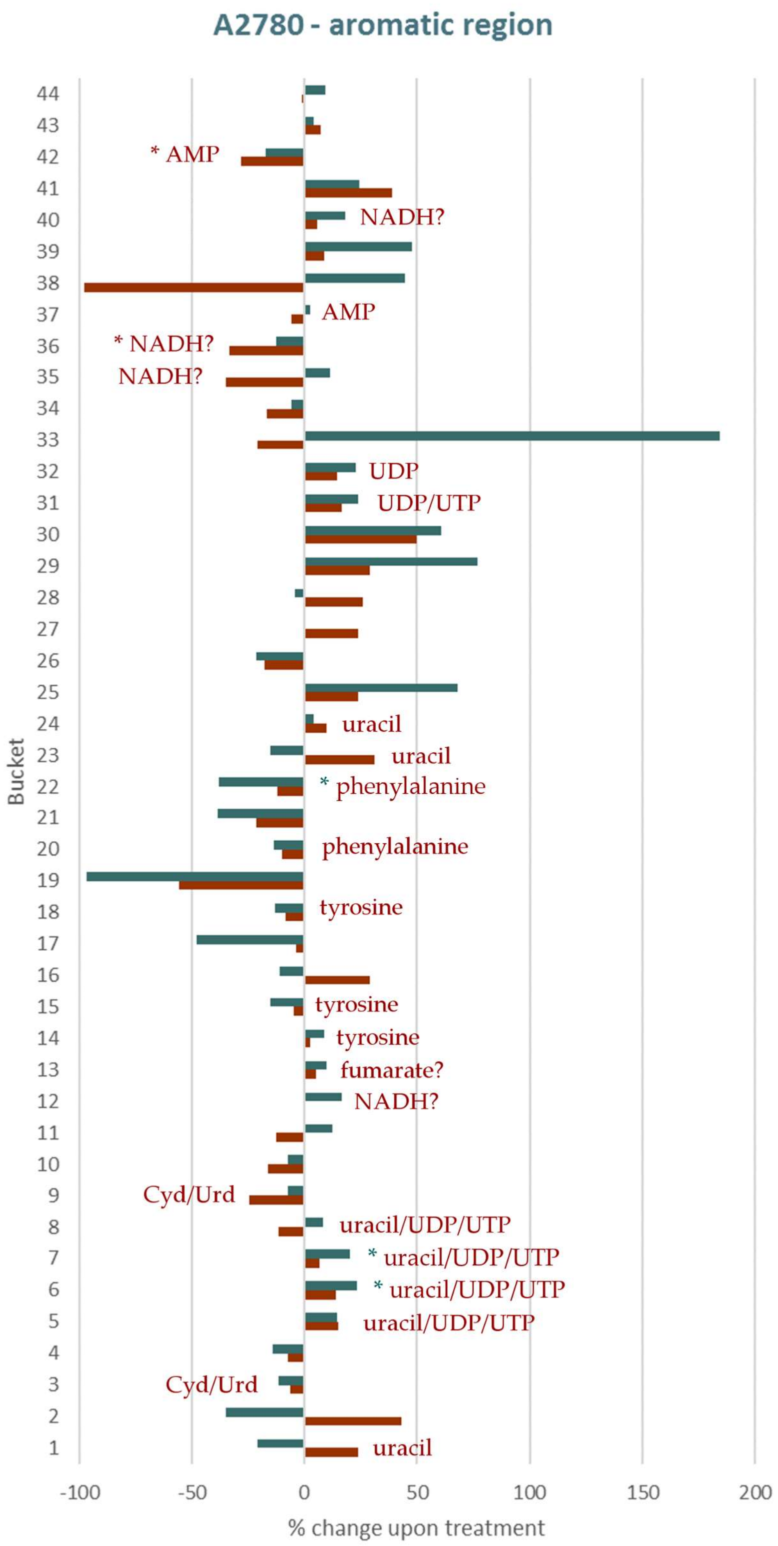

Figure S18. Percental change in the aromatic buckets in A2780 cells: The increasing/decreasing level of each bucket compared to the control in cells treated with 0.015 µM DiRu-1 (red) and 0.03 µM DiRu-1 (turquoise) are expressed in percentage. * p-value < 0.05

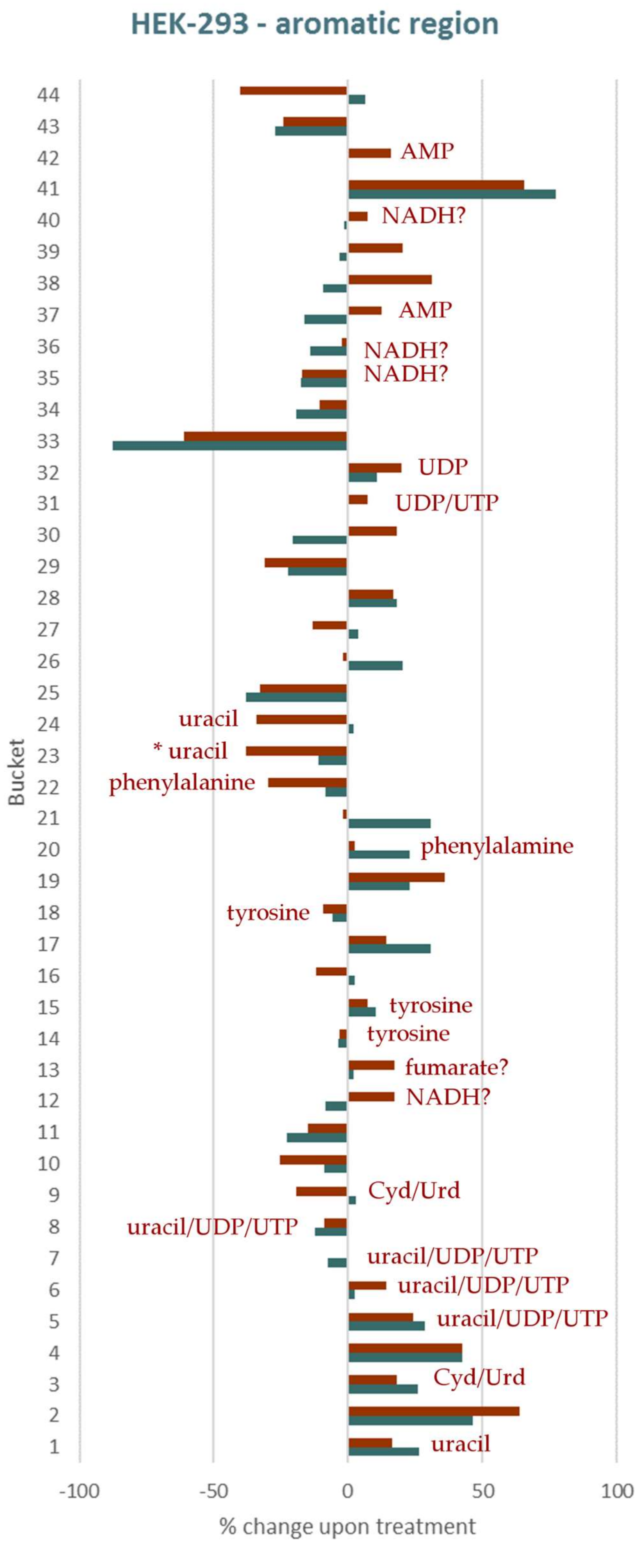

Figure S19. Percental change in the aromatic buckets in HEK-293 cells: The increasing/decreasing level of each bucket compared to the control in cells treated with 0.015 µM DiRu-1 (turquoise) and 0.03 µM DiRu-1 (red) are expressed in percentage. * p-value < 0.05

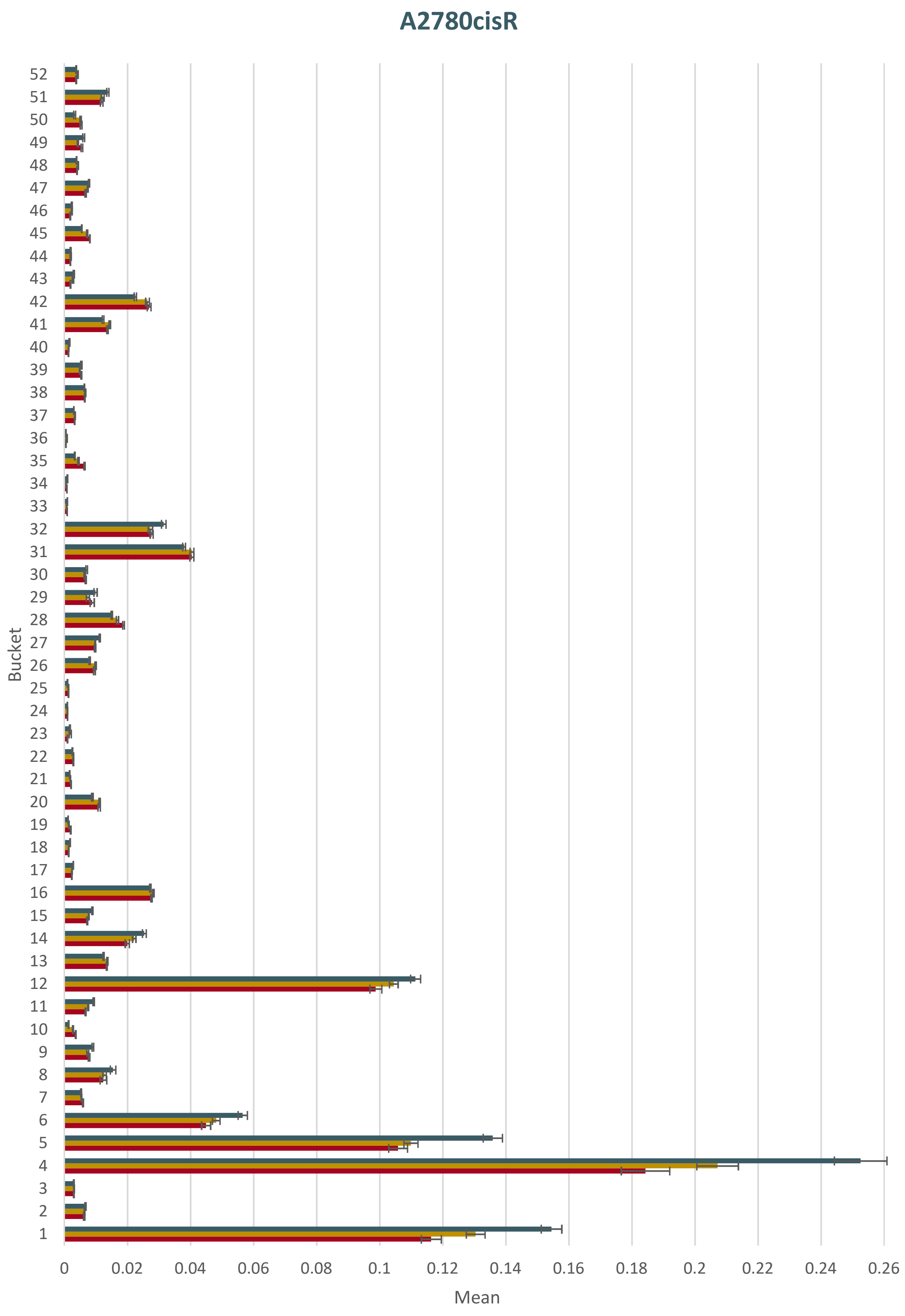

Figure S20. Comparison of bucket means for aliphatic region of A2780cisR: Control (blue), treated with 0.015 µM DiRu-1 (yellow), and 0.03 µM DiRu-1 (red). Error bars show standard error.

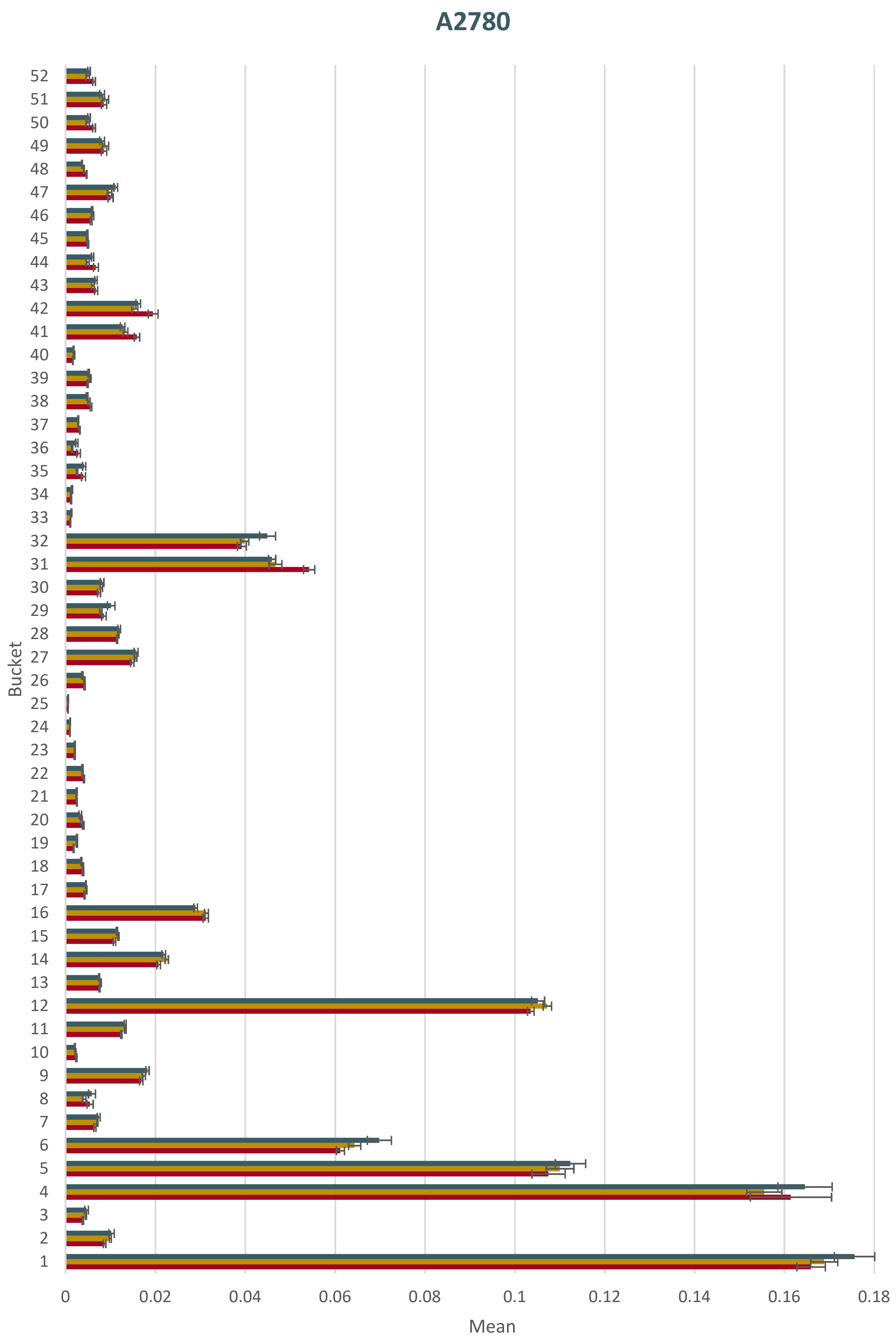

Figure S21. Comparison of bucket means for aliphatic region of A2780: Control (blue), treated with 0.015 μM DiRu-1 (yellow), and 0.03 μM DiRu-1 (red). Error bars show standard error.

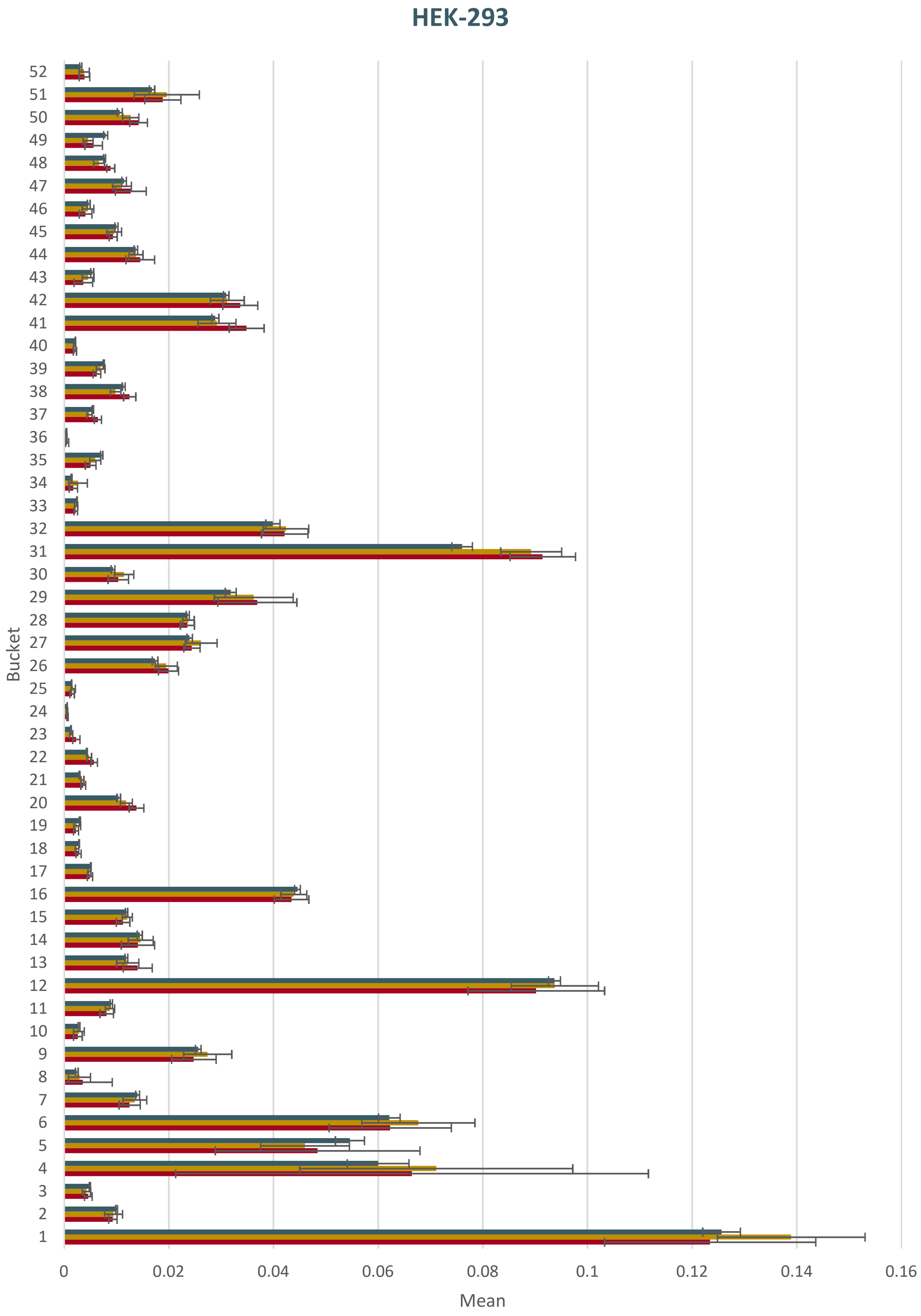

Figure S22. Comparison of bucket means for aliphatic region of HEK-293: Control (blue), treated with 0.015 μM DiRu-1 (yellow), and 0.03 μM DiRu-1 (red). Error bars show standard error.

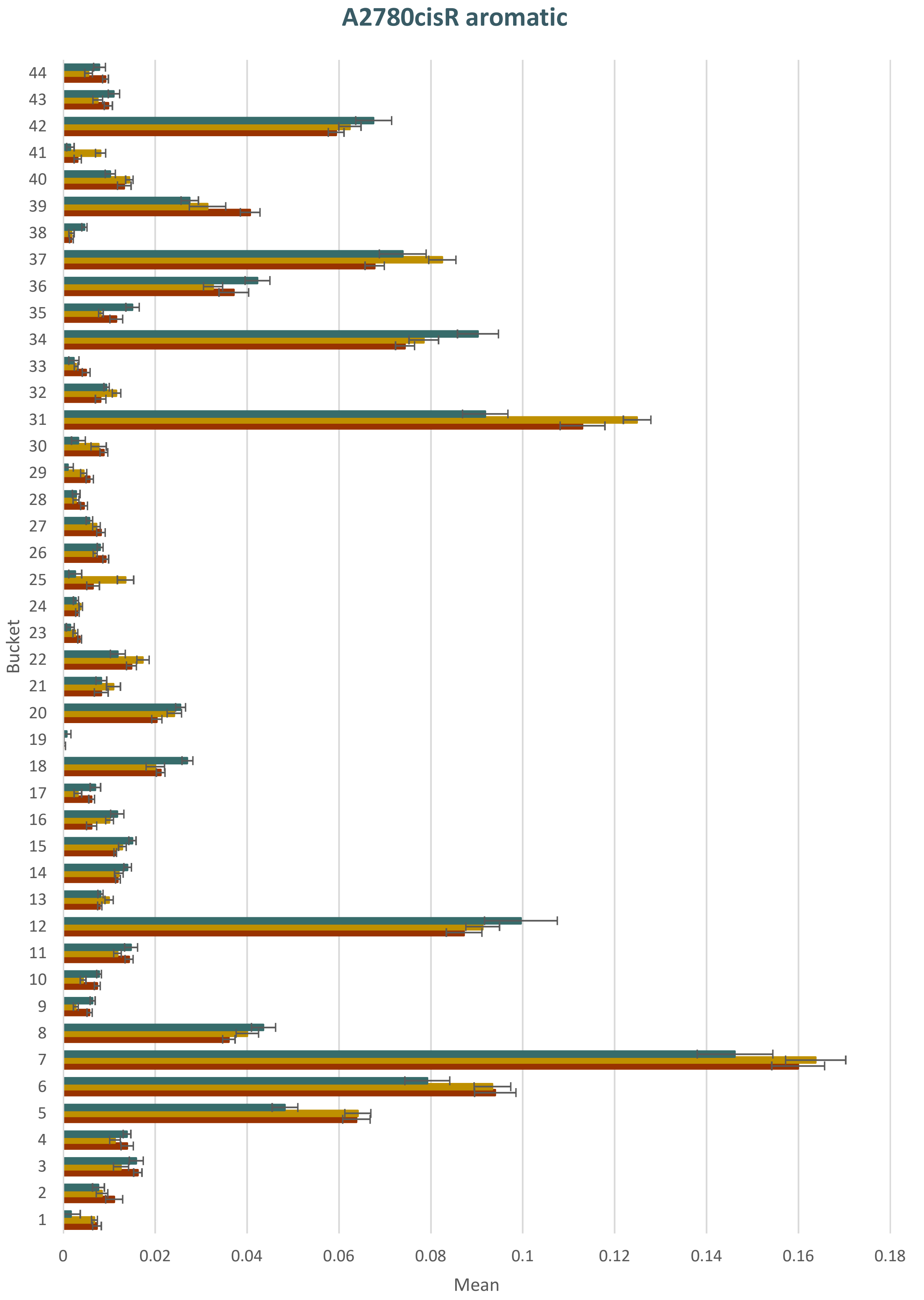

Figure S23. Comparison of bucket means for aromatic region of A2780cisR: Control (blue), treated with 0.015 µM DiRu-1 (yellow), and 0.03 µM DiRu-1 (red). Error bars show standard error.

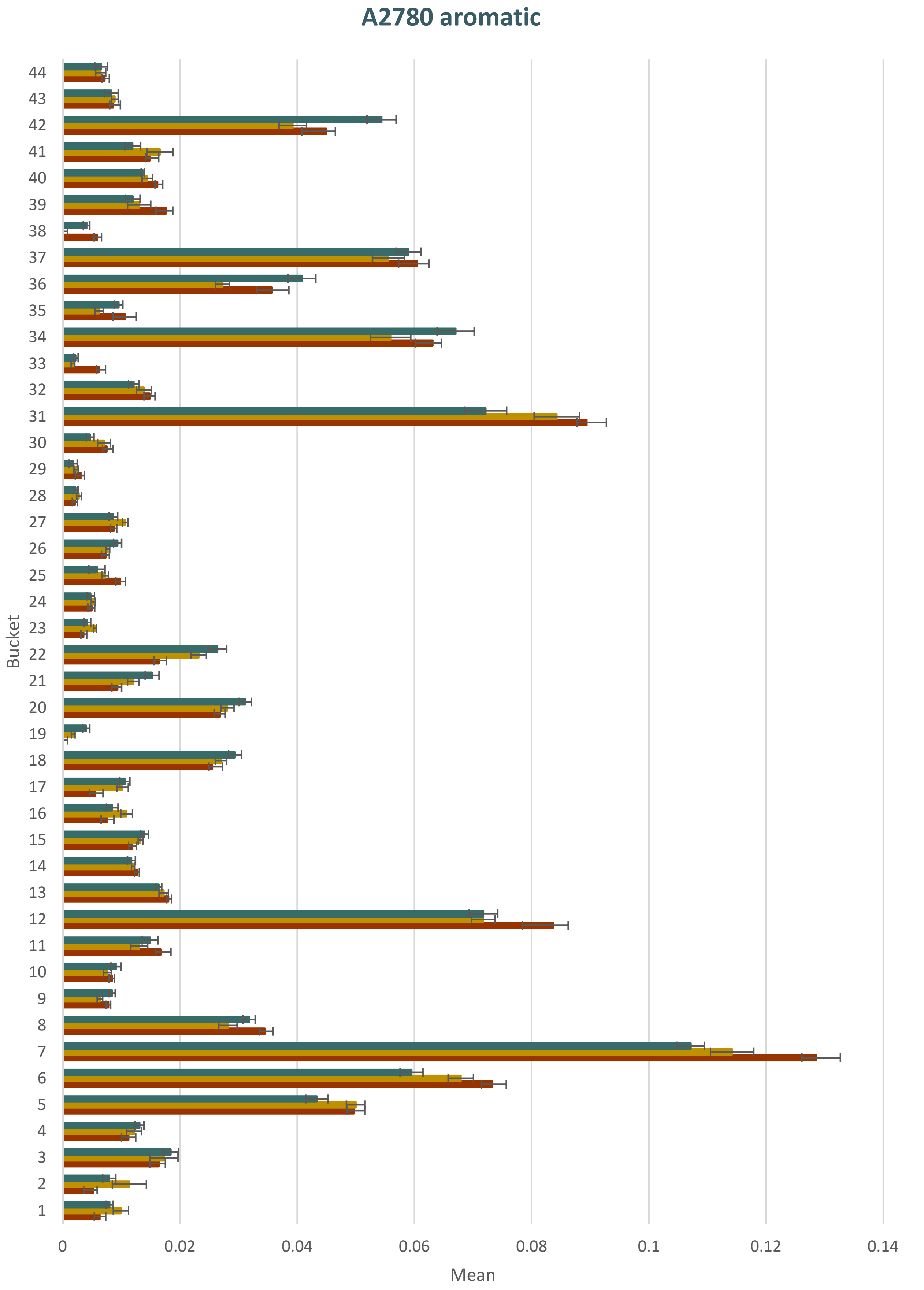

Figure S24. Comparison of bucket means for aromatic region of A2780: Control (blue), treated with 0.015 μM DiRu-1 (yellow), and 0.03 μM DiRu-1 (red). Error bars show standard error.

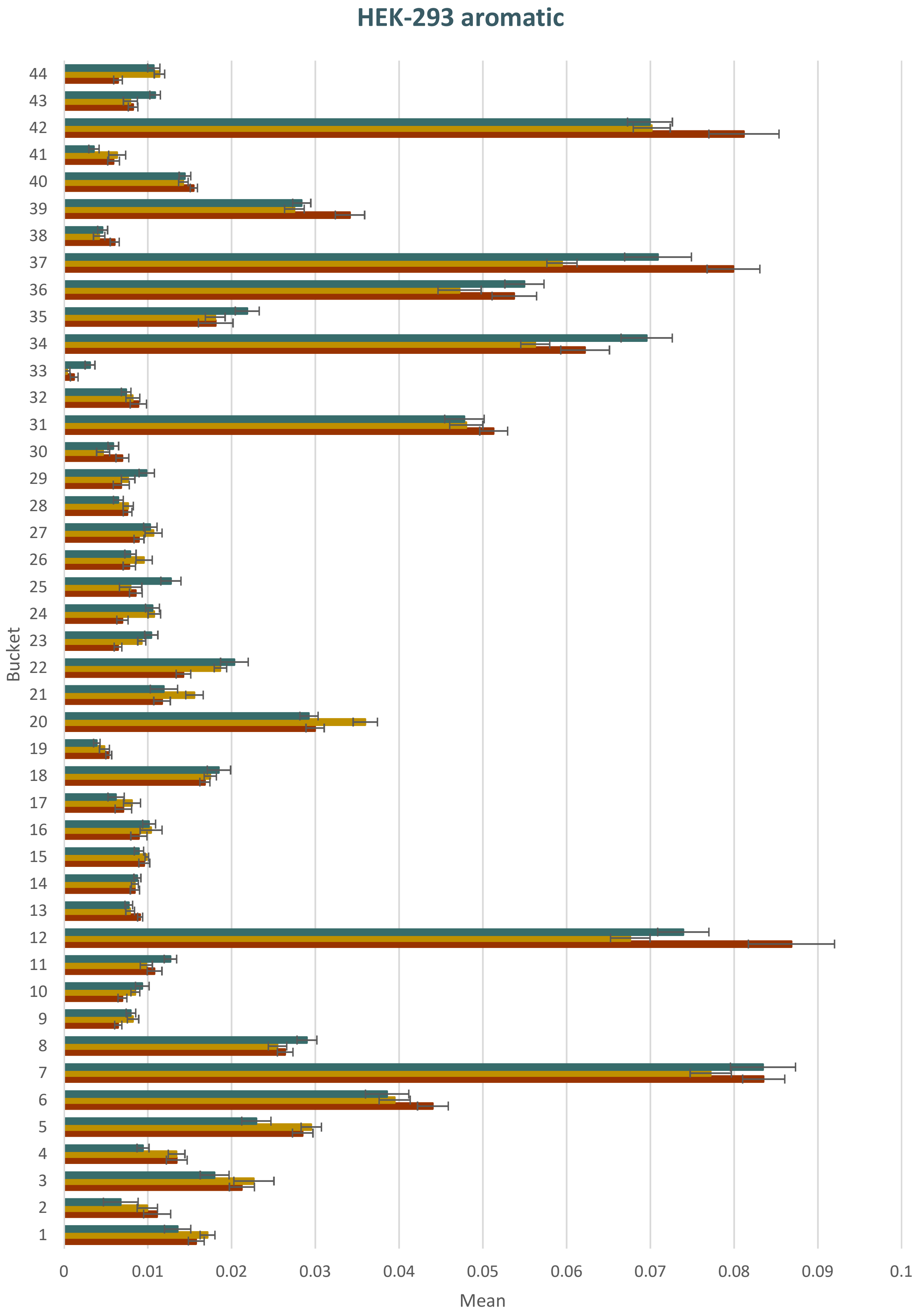

Figure S25. Comparison of bucket means for aromatic region of HEK-293: Control (blue), treated with 0.015 μM DiRu-1 (yellow), and 0.03 μM DiRu-1 (red). Error bars show standard error.
